# STING activation reprograms the microenvironment to sensitize NF1-related malignant peripheral nerve sheath tumors for immunotherapy

**DOI:** 10.1172/JCI176748

**Published:** 2024-03-19

**Authors:** Bandarigoda N. Somatilaka, Laasya Madana, Ali Sadek, Zhiguo Chen, Sanjay Chandrasekaran, Renee M. McKay, Lu Q. Le

**Affiliations:** 1 Department of Dermatology,; 2 Simmons Comprehensive Cancer Center,; 3 Department of Internal Medicine, Division of Hematology/Oncology,; 4 University of Texas Southwestern Comprehensive Neurofibromatosis Clinic,; 5 Hamon Center for Regenerative Science and Medicine, and; 6 O’Donnell Brain Institute, University of Texas Southwestern Medical Center at Dallas, Dallas, Texas, USA.; 7 Department of Dermatology, University of Virginia School of Medicine, Charlottesville, Virginia, USA.

**Keywords:** Oncology, Therapeutics, Cancer immunotherapy, Immunotherapy

## Abstract

Neurofibromatosis type 1 (NF1) is caused by mutations in the *NF1* gene that encodes neurofibromin, a RAS GTPase–activating protein. Inactivating *NF1* mutations cause hyperactivation of RAS-mediated signaling, resulting in the development of multiple neoplasms, including malignant peripheral nerve sheath tumors (MPNSTs). MPNSTs are an aggressive tumor and the main cause of mortality in patients with NF1. MPNSTs are difficult to resect and refractory to chemo- and radiotherapy, and no molecular therapies currently exist. Immune checkpoint blockade (ICB) is an approach to treat inoperable, undruggable cancers like MPNST, but successful outcomes require an immune cell–rich tumor microenvironment. While MPNSTs are noninflamed “cold” tumors, here, we converted MPNSTs into T cell–inflamed “hot” tumors by activating stimulator of IFN genes (STING) signaling. Mouse genetic and human xenograft MPNST models treated with a STING agonist plus ICB exhibited growth delay via increased apoptotic cell death. This strategy offers a potential treatment regimen for MPNSTs.

## Abstract 2

A STING agonist turns noninflamed &quot;cold&quot; malignant peripheral nerve sheath tumors into T cell&ndash;inflamed &quot;hot&quot; tumors, making them amenable to immune checkpoint blockade in mice.

## Graphical abstract

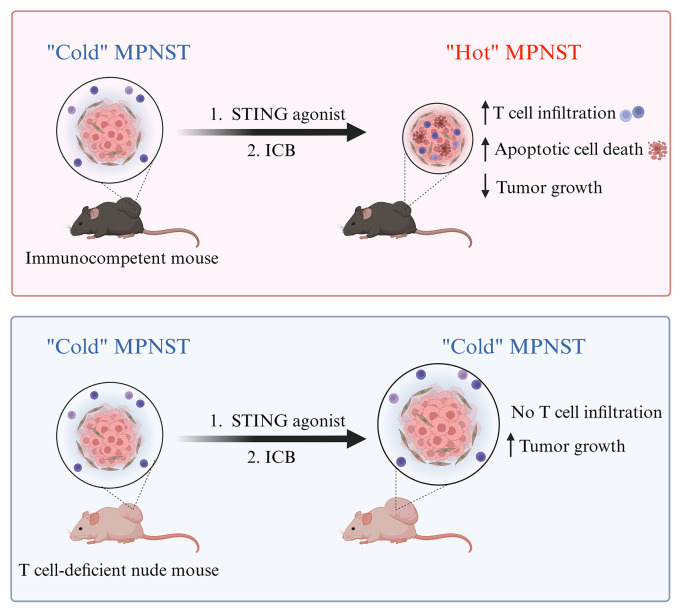

## Introduction

Neurofibromatosis type 1 (NF1) affects 1 in 3,000 individuals. A hallmark feature of NF1 is the development of benign cutaneous and plexiform neurofibromas (pNFs) that arise in the skin and peripheral nerve plexuses, respectively. In up to 10% of patients with NF1, benign pNFs undergo transformation into malignant peripheral nerve sheath tumors (MPNSTs), which are highly aggressive and are the leading cause of death in patients with NF1 (1).

NF1 is associated with inactivating mutations in the *NF1* gene that encodes neurofibromin, a RAS GTPase–activating protein. Neurofibromin binds to the GTP-bound active form of RAS and enhances GTPase activity, negatively regulating downstream signaling. As a result, inactivating mutations in *NF1* activate multiple effector cascades including the RAS/RAF/MEK/ERK and PI3K/AKT/mTOR pathways (2). Inhibition of RAS and RAS-activated downstream signaling pathways have been explored as treatments for MPNST, however, these efforts have been unsuccessful, yielding no significant improvement in survival for patients with MPNST in clinical trials (3–5). The MEK inhibitor selumetinib is currently the only FDA-approved drug for the treatment of NF1-associated inoperable pNF, but it is not effective against MPNST (6–9). To date, pharmacologic targeting of dysregulated signaling pathways remains an unsuccessful strategy to treat MPNST. Surgical resection, therefore, remains the primary treatment for MPNST, although achieving complete tumor removal is often challenging, given the large size of the tumor and/or its proximity to nerves (10, 11). Additionally, MPNSTs often metastasize, and patients have a high tendency to relapse following tumor resection. MPNSTs are also refractory to chemotherapy and radiotherapy, leading to dismal survival rates for patients with MPNST (12–15). Thus, new, effective treatment strategies for MPNST are desperately needed.

Cancer cells use a variety of mechanisms to escape destruction by the host immune system. One way is by hijacking immune checkpoint control mechanisms that serve to avoid collateral damage during a normal immune response. The programmed cell death protein 1 (PD-1) pathway limits T cell effector functions within tissues. By expressing its ligand PD-L1, tumor cells block T cell–mediated antitumor immune responses in the tumor microenvironment (TME) (16). Immune checkpoint blockade (ICB) therapy is a strategy in cancer immunotherapy that disrupts these ligand-receptor interactions, reprogramming a patient’s immune system to target inoperable, undruggable tumors (17) by inhibiting ligand-receptor interactions used by cancer cells to escape immune destruction. The use of monoclonal antibodies to disrupt the interaction between PD-1 and PD-L1 is a widely accepted ICB therapy, however, having a T cell–enriched TME is critical for ICB therapy success (17). Unfortunately, MPNSTs are non–T cell–inflamed or “cold” tumors and are therefore not likely to elicit an antitumor immune response to checkpoint inhibition (18–20). It has been shown that some MPNSTs have more prevalent PD-L1 expression than do normal nerves, benign neurofibromas, or schwannomas (19), whereas another study reported similar levels of PD-L1 expression in MPNST and benign NF–related tumors (21). A more recent study showed that PD-L1 was significantly elevated in the sera of NF1 patients with MPNSTs compared with NF1 patients without MPNSTs, suggesting a positive correlation between PD-L1 expression and MPNST progression (22). Therefore, increasing intratumoral T cell density, along with immune checkpoint inhibitor treatment, could generate T cell–mediated antitumor responses in MPNST.

The discovery of how the cyclic GMP-AMP synthase/stimulator of interferon (IFN) genes/IFN (cGAS/STING/IFN) pathway augments antitumor immunity via enrichment of an immune-suppressive TME may lead to further breakthroughs in cancer immunotherapy (23, 24). The cGAS enzyme binds to naked dsDNA and undergoes conformational changes that allow it to convert ATP and GTP into 2′3′-cyclic GMP-AMP (cGAMP). cGAMP functions as a second messenger that binds to its ER-resident adaptor protein STING. cGAMP binding induces a conformational change in STING that exposes the C-terminal tail for TANK-binding kinase 1 (TBK1) binding and activation. TBK1 phosphorylates IFN regulatory factor 3 (IRF3), which induces type I IFNs. Type I IFNs bind to the type I IFN receptor, activating a signaling cascade leading to the expression of IFN-stimulated genes. STING also activates inhibitor of NF-κB (IκB) kinase (IKK) and subsequently NF-κB for proinflammatory cytokine induction. Activation of the STING/IFN axis in tumor cells promotes antiproliferative and immunomodulatory activities including enhancement of T cell infiltration into the TME (25, 26). Therefore, activation of the STING/IFN pathway could convert cold MPNSTs into hot tumors.

Here, we report that treatment of a genetic, spontaneous mouse MPNST model with a STING agonist converted the TME from cold to hot, as shown by the intratumoral infiltration of T cells. Treating this in vivo mouse model with both a STING agonist and ICB resulted in apoptosis of tumor cells and inhibition of tumor growth. Furthermore, STING activation followed by ICB caused a much-accelerated, complete regression of human tumors in a xenograft model of MPNST. These studies leveraging our preclinical MPNST models support the idea of testing the combination of a STING agonist with ICB as a treatment strategy for NF1 patients with MPNST.

## Results

### MPNSTs are cold tumors but do express PD-L1.

**Figure 1:**
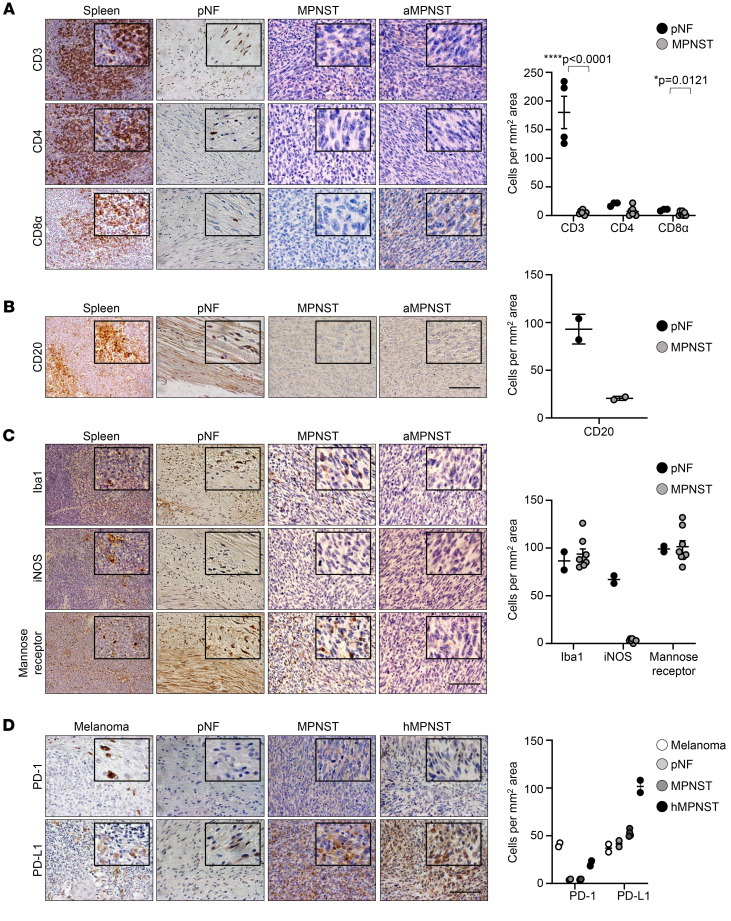
Characterization of the immune microenvironment of MPNST. (**A**–**D**) Paraffin sections of murine spleen, murine pNF (harvested from *Sox10-CreERT* *Nf1^fl/fl^* mice induced with tamoxifen), murine MPNST (from *cisNP* mice), and MPNST allografts in athymic nude mice (aMPNST) were stained with antibodies against CD3, CD4, and CD8α (**A**); CD20 (**B**); and Iba1, iNOS, and the mannose receptor (**C**). (**D**) Paraffin sections of human melanoma, murine pNFs, murine MPNSTs, and human MPNSTs (hMPNST) were stained with antibodies against PD-1 and PD-L1. Sections in **A**–**D** were counterstained with hematoxylin (blue), and the respective cell counts for **A**–**D** are shown on the right. Data indicate the mean ± SEM (**A**) and the mean ± SD (**B**–**D**). *P < 0.0 and ****P < 0.0001, by 2-tailed *t* test with respect to pNF (**A**). Scale bars: 50 μm. Original magnification, ×80 (enlarged insets).

A spontaneous mouse model of MPNST was generated by recombination of *Nf1*- and *p53*-null alleles in *cis* on chromosome 11 (27, 28). As a result of spontaneous loss of the WT *Nf1* and *p53* alleles, *cisNf1^+/–^* *p53^+/–^* (hereafter referred to as *cisNP*) mice develop a variety of sarcomas including MPNST (*cis*MPNST) between 3 and 7 months of age. Human MPNSTs have been reported to be cold tumors lacking T cell infiltration (18, 19, 29). To confirm that the MPNSTs that develop in this *cisNP* mouse model were also noninflamed tumors, we performed IHC using antibodies against various immune cell types. As has been reported for human MPNST, we found that *cis*MPNSTs contained few T cells (including CD3^+^ T cells, CD4^+^ T helper [Th] cells, and CD8α^+^ cytotoxic T cells) (Figure 1A) and few B cells (CD20^+^) (Figure 1B) compared with spleen and pNFs. They expressed macrophages (Iba1^+^), including few M1 macrophages (iNOS^+^) and more M2 macrophages (mannose receptor^+^), compared with pNFs (Figure 1C). MPNSTs that developed in allograft mice, in which MPNST cells were harvested from *cisNP* mice and implanted into athymic nude mouse hosts, were also negative for CD3, CD4, and CD8α expression (Figure 1A), which was expected and served as a control, since nude mice do not have a normal immune system and lack T cells (30). Thus, *cis*MPNSTs were cold tumors lacking an inflamed TME.

PD-1 is usually expressed by immune cells to terminate an immune response and avoid collateral tissue damage (31). Therefore, a lack of PD-1 expression in *cis*MPNSTs would be consistent with the observed lack of T cell infiltration into the MPNST microenvironment. IHC for PD-1 expression confirmed that these tumors infrequently expressed PD-1 (Figure 1D). Interestingly, we found that, like human MPNSTs, mouse *cis*MPNSTs contained PD-L1–expressing cells (Figure 1D and Supplemental Figure 1A; supplemental material available online with this article; https://doi.org/10.1172/JCI176748DS1), thereby qualifying MPNST as a candidate for ICB targeting.

### STING agonist treatment activates the STING pathway in MPNSTs.

The cytosolic DNA–sensing enzyme cGAS binds to dsDNA and initiates a cascade of events leading to the production of type I IFNs and proinflammatory cytokines and chemokines. This cytokine response presumably recruits immune cell infiltration into the TME. As such, a number of STING agonists have been developed for testing as potential immune boosters (32). Preclinical studies using mouse tumor models have assessed the efficacy of STING agonists in triggering the cGAS/STING/IFN axis and shown that these agonists increase innate immunity and produce a CD8^+^ T cell–rich environment (33–35). However, this approach has not been tested in MPNSTs.

We hypothesized that treating the *cisNP* MPNSTs with a STING agonist would result in the expression of cytokines and chemokines that would recruit T cells into the tumor. To test this, we used 2 different commercially available STING agonists — synthetic dinucleotide ADU-S100 (33–35) and synthetic non-nucleotide STING agonist 3 (SA3) (36). We first tested ADU-S100 on 2 different MPNST cell lines: a mouse MPNST cell line derived from *Nf1*- and *p53*-null skin progenitor cells (HTS-Luc MPNST) and *cis*MPNST cells derived from *cisNP* mice. Cells were treated for 8, 18, 24, or 48 hours and then harvested for Western blot analysis, which showed increased expression of phosphorylated IRF (p-IRF) and p–NF-ĸB, indicating STING pathway activation after 8 hours of ADU-S100 treatment (Supplemental Figure 1, B and C). Quantitative reverse transcription real-time PCR (qRT-PCR) had the highest expression of cytokine/chemokine genes 8 hours after ADU-S100 treatment (Supplemental Figure 1D), demonstrating that ADU-S100 treatment could activate STING/IFN signaling in MPNST cells.

**Figure 2:**
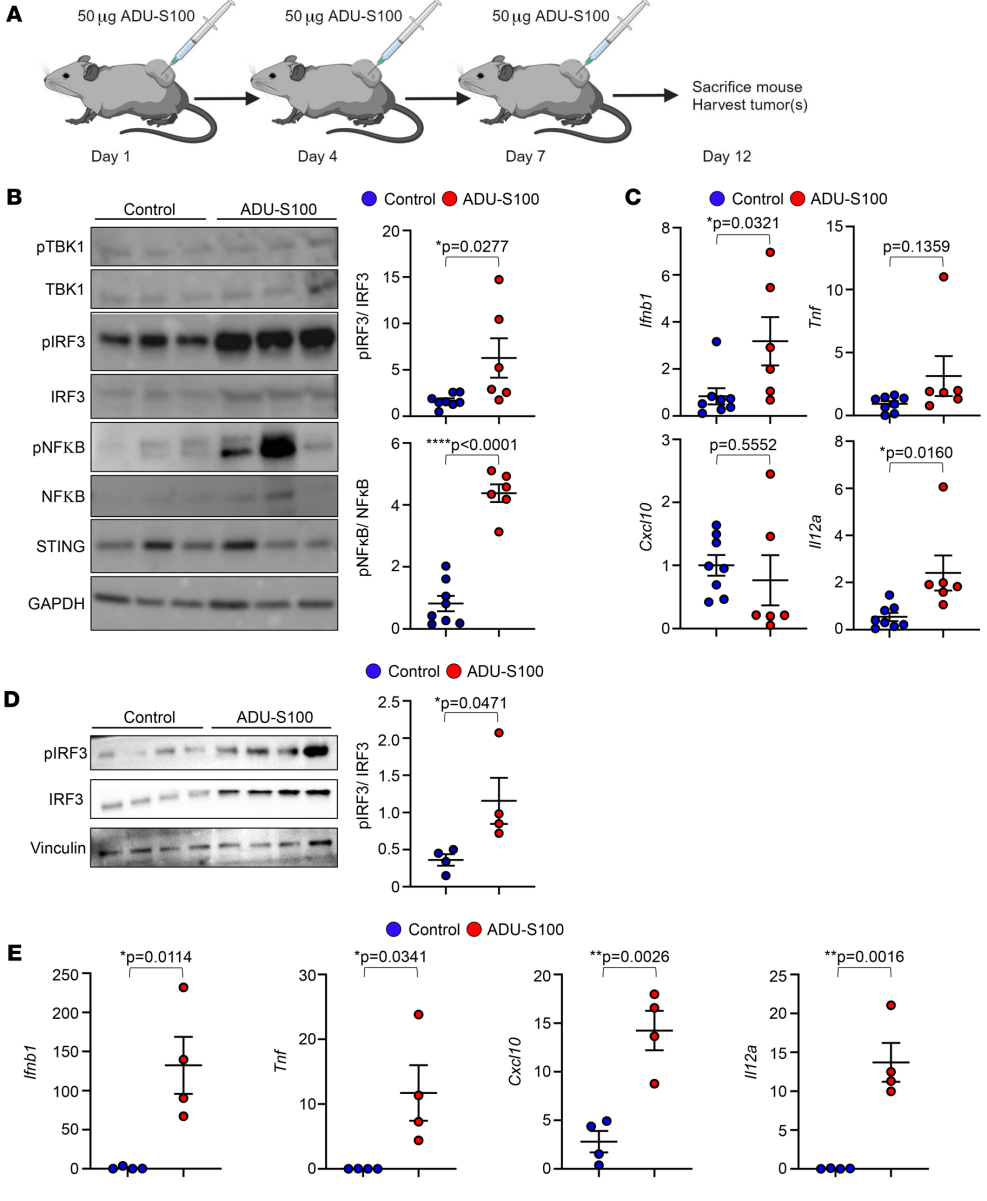
ADU-S100 treatment of *cisNP* mice activates the STING pathway in tumors. (**A**) Schema of the ADU-S100 treatment protocol. (**B**) Western blot analysis for expression of the indicated proteins in MPNSTs harvested from *cisNP* mice treated with vehicle control (*n* = 8) or ADU-S100 (*n* = 6). Quantified protein band intensities are shown graphically on the right. (**C**) PCR analysis of the fold change in cytokine gene expression (*Ifnb1*, *Tnf*, *Cxcl10*, and *Il12a*) in *cis*MPNSTs harvested from control-treated (*n* = 8) and ADU-S100–treated (*n* = 6) mice. (**D**) Western blot analysis for expression of the indicated proteins in MPNSTs harvested from *cisNP* mice treated with vehicle control (*n* = 4) or ADU-S100 (*n* = 4) for 24 hours. Quantified protein band intensities are shown graphically on the right. (**E**) PCR analysis of fold change in cytokine gene expression (*Ifnb1*, *Tnf*, *Cxcl10*, and *Il12a*) in *cis*MPNSTs harvested from control-treated (*n* = 4) and ADU-S100–treated (*n* = 4) mice 24 hours after treatment. Data are presented as the mean ± SEM. *P < 0.05, **P < 0.01, and ****P < 0.0001, by 2-tailed *t* test versus vehicle control.

We next tested STING agonists for activity in vivo: MPNSTs that developed in *cisNP* mice were injected either intratumorally with ADU-S100 or intraperitoneally with SA3 and monitored for 12 days (Figure 2A, Supplemental Figure 2A, and Table 1). On day 12, the tumors were harvested and analyzed for activation of the STING pathway. We observed that markers of STING pathway activation such as p-IRF3 and p–NF-ĸB were indeed upregulated in both the ADU-S100– and SA3-treated tumors compared with vehicle controls (Figure 2B and Supplemental Figure 2B), as was expression of the proinflammatory cytokines/chemokines *Ifnb1*, *Tnf*, *Cxcl10*, and *Il12a* (Figure 2C and Supplemental Figure 2C). Although the expression of some of the genes we analyzed showed a statistically significant increase upon ADU-S100 treatment, the expression of others was increased but did not reach statistical significance (Figure 2C). This could be because upregulation of STING signaling following STING agonist treatment was transient, as evident from the in vitro experiments. As a result, our timing of analysis — 5 days after the last treatment (day 12 of the treatment scheme; Figure 2A) — could have been too late to capture the more transitory changes in STING activation. To assess the temporal effect on cytokine expression, we harvested *cis*MPNSTs earlier, at 24 hours after ADU-S100 treatment, and evaluated phosphorylated protein expression and target gene expression. At 24 hours, we saw statistically significant upregulation of the STING signaling pathway (Figure 2, D and E).

### STING activation promotes T cell infiltration into MPNST in vivo.

**Figure 3:**
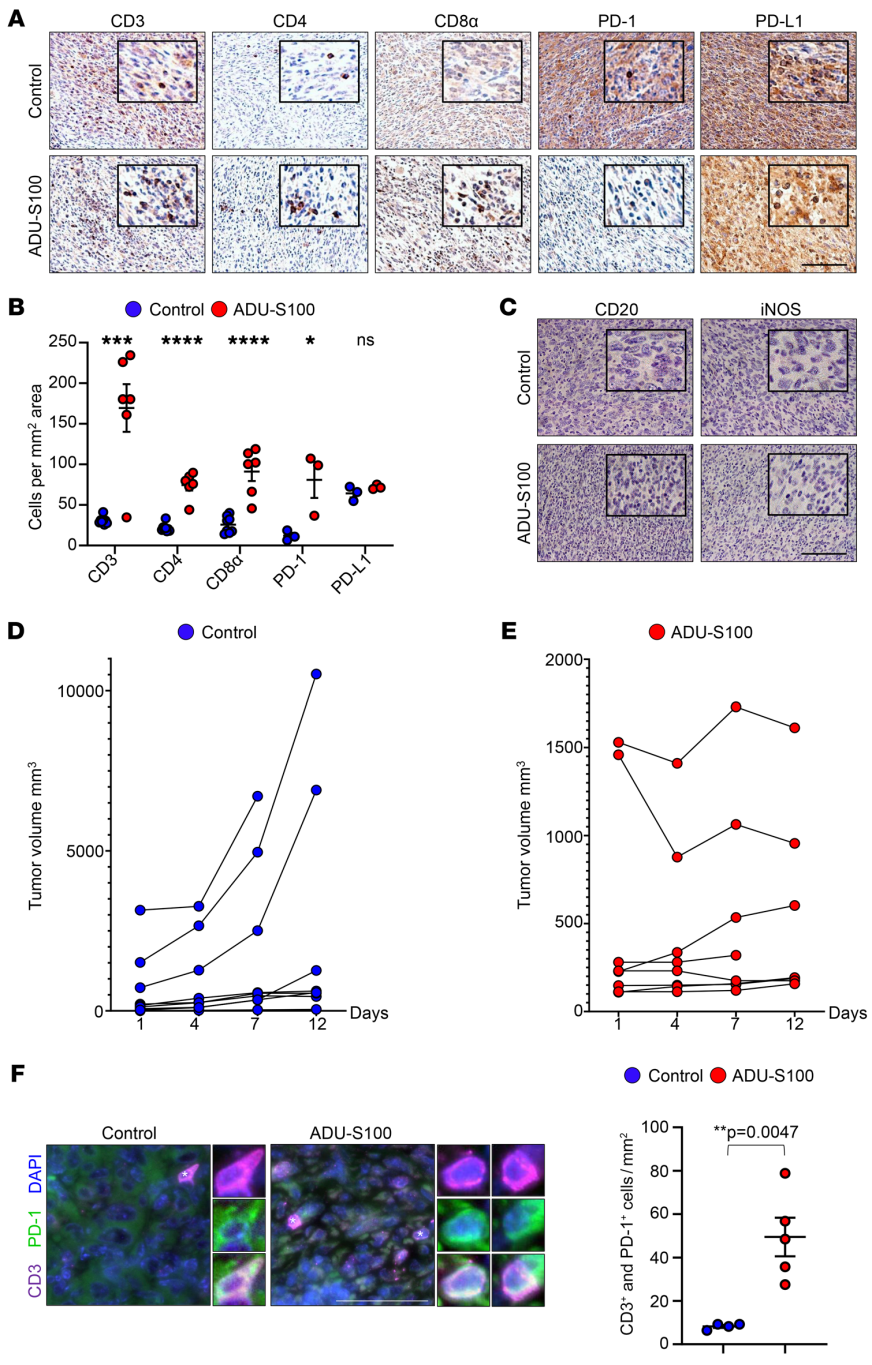
STING activation in MPNST increases T cell infiltration and impedes tumor growth. (**A**) Paraffin sections of MPNSTs harvested from vehicle-treated or ADU-S100–treated *cisNP* mice were stained with antibodies against CD3, CD4, CD8α, PD-1, and PD-L1 and (**B**) quantified. (**C**) The same sections were also stained for CD20 and iNOS. (**D** and **E**) Tumor volume change with time in response to indicated treatments. (**F**) Coimmunostaining for CD3 and PD-1 with quantification. Cells marked with asterisks in each panel are magnified and shown adjacently. Control, *n* = 4; ADU-S100, *n* = 5. Scale bars: 50 μm. Original magnification, ×80 (enlarged insets in **A** and **C**) and ×160 (enlarged insets in **F**). Data are presented as the mean ± SEM. *P < 0.05, **P < 0.01, ***P < 0.001, and ****P < 0.0001, by unpaired, 2-tailed *t* test versus vehicle control. See Methods for a detailed description of the staining methodology.

To determine whether activation of the STING pathway by ADU-S100 or SA3 was sufficient to recruit T cells into the *cis*MPNST TME, we performed IHC for T cell markers. IHC revealed that treatment with the STING agonists increased infiltration of CD3^+^ T cells, CD4^+^ Th cells, and CD8^+^ cytotoxic T cells, as well as PD-1–expressing cells, into the tumor (Figure 3, A and B, and Supplemental Figure 2, D and E). The number of PD-L1–expressing cells, however, was unchanged (Figure 3, A and B, and Supplemental Figure 2, D and E). IHC for the B cell marker CD20 and the M1 macrophage marker iNOS showed no increase in the presence of either of these immune cell types (Figure 3C).

Together, these data demonstrate that treatment with the STING agonist activated the STING pathway in our *cisNP* mice and promoted T cell infiltration into the tumor, thus transforming cold MPNSTs into hot tumors.

### STING activation by STING agonists impedes MPNST growth.

To determine whether STING activation alone had any effect on MPNST growth, we monitored tumor growth of STING agonist–treated tumors compared with vehicle-treated tumors. We found that treatment with either ADU-S100 alone or SA3 alone resulted in slower tumor growth, indicating that STING activation and subsequent recruitment of immune cells to the tumor can impede tumor growth (Figure 3, D and E, and Supplemental Figure 2F). However, STING agonist treatment alone did not cause complete regression of tumors. Costaining with antibodies against CD3 and PD-1 demonstrated that a subset of PD-1–expressing cells were indeed T cells (Figure 3F). Since the tumor cells express PD-L1 (Figure 1D, Figure 3, A and B, and Supplemental Figure 2, D and E), it was possible that interaction of PD-L1 with PD-1 expressed on infiltrating T cells could cause immune escape of the tumor cells, leading to persistence of the tumor (16). T cell–intrinsic STING signaling has been shown to promote Treg induction (37). A recent report also showed that Foxp3^+^ Tregs are abundant in human MPNSTs (38). Therefore, we investigated whether Foxp3^+^ T cells were present in the *cis*MPNSTs (Supplemental Figure 3, A and B). Compared with the WT mouse spleen, vehicle-treated *cis*MPNSTs showed few Foxp3^+^ cells. This was unaltered upon ADU-S100 treatment (Supplemental Figure 3A). Furthermore, immunoblot analysis did not show a significant difference in FOXP3 protein levels between vehicle- and ADU-S100–treated tumors (Supplemental Figure 3B).

As a control, we generated *cis*MPNSTs in athymic nude mice by subcutaneously implanting tumors harvested from *cisNP* mice into athymic mice (Supplemental Figure 4A). These allograft *cis*MPNSTs in athymic mice continued to grow despite ADU-S100 treatment (Supplemental Figure 4, B and C), suggesting that the immune inflammation resulting from STING activation was mediated by T cells in the context of MPNST. Here, we confirmed that the STING pathway was activated 24 hours after ADU-S100 treatment to rule out the possibility of non-upregulation of the pathway resulting in tumors responding similarly in the 2 experimental groups (Supplemental Figure 4, D and E).

Because of the close proximity to the *NF1* gene, somatic *TP53* mutations are frequent in human MPNSTs (39, 40). Although the *cisNP* model is similar to such human NF1-associated MPNSTs, it could also present complications in a study such as ours, as it is reported that mutations in p53 can affect immune cell function (41). Therefore, to overcome potential limitations of the *cisNP* model, in parallel, we treated a different mouse MPNST model *— PLP-CreERT2* *Nf1^fl/fl^* *p53^fl/fl^* mice (hereafter referred to as conditional MPNST) *—* with ADU-S100 (Supplemental Figure 5A). In this model, *Nf1* and *p53* are conditionally deleted in Schwann cell precursors, thus providing spatiotemporal control over MPNST generation. Once mice were 1 week old, we subcutaneously injected 4-hydroxytamoxifen (dissolved in 100% ETOH at 4 mg/mL, 40 μg per pup) to induce conditional deletion of *Nf1* and *p53*. We let tumors develop until they were at least 5 mm in diameter and then treated them with ADU-S100 or vehicle. As in the case of *cis*MPNST, growth of these tumors was significantly delayed upon ADU-S100 treatment but had not completely regressed (Supplemental Figure 5, B and C).

### Combination treatment of cisNP mice with a STING agonist plus ICB slows tumor growth, increases T cell infiltration, and promotes apoptosis in MPNSTs.

**Figure 4:**
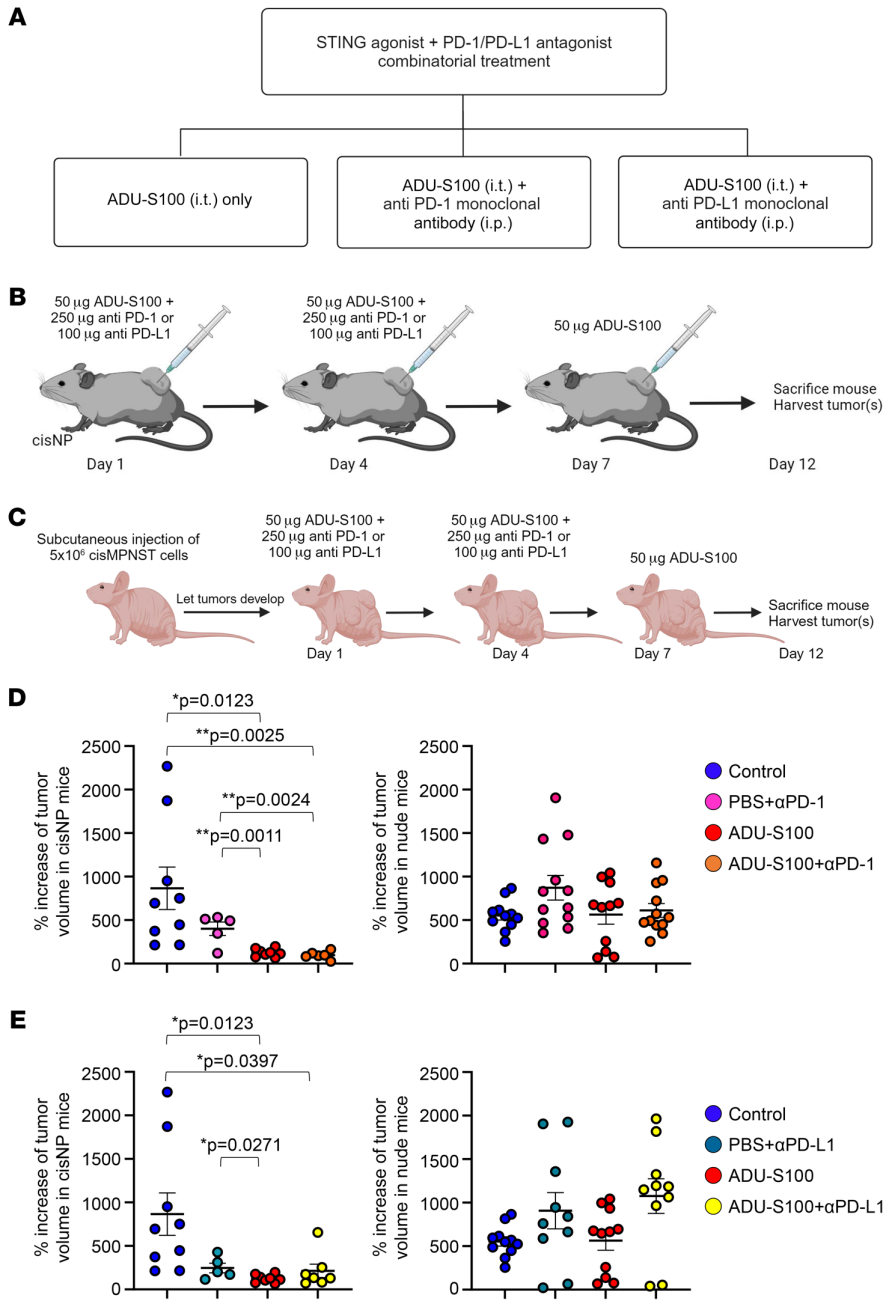
Combination treatment of *cisNP* mice with a STING agonist plus ICB delays tumor growth. (**A**) Treatment arms for STING activation followed by the ICB study. i.t., intratumoral. (**B**) Schema of drug treatment for STING activation and ICB in *cisNP* mice. (**C**) Schema of STING activation and ICB in nude mice. (**D** and **E**) Percentage of increase in tumor volume in *cisNP* mice and nude mice given the indicated treatments. Control, *n* = 9; ADU-S100, ADU-S100 plus anti–PD-1 (αPD-1), *n* = 6; PBS plus anti–PD-1, *n* = 6; PBS plus anti–PD-L1, *n* = 5; ADU-S100 plus anti–PD-L1, *n* = 7 for *cisNP* mice. Control, *n* = 11; ADU-S100, *n* = 11; ADU-S100 plus anti–PD-1, *n* = 12; PBS plus anti–PD-1, *n* = 12; PBS plus anti–PD-L1, *n* = 10; ADU-S100 plus anti–PD-L1, *n* = 10 for nude mice. The same data sets for control and ADU-S100 treatments in **D** are shown in the respective graphs in **E** for clarity and ease of comparison. Data are presented as the mean ± SEM. *P < 0.05 and **P < 0.01, by Tukey’s multiple-comparison test.

**Figure 5:**
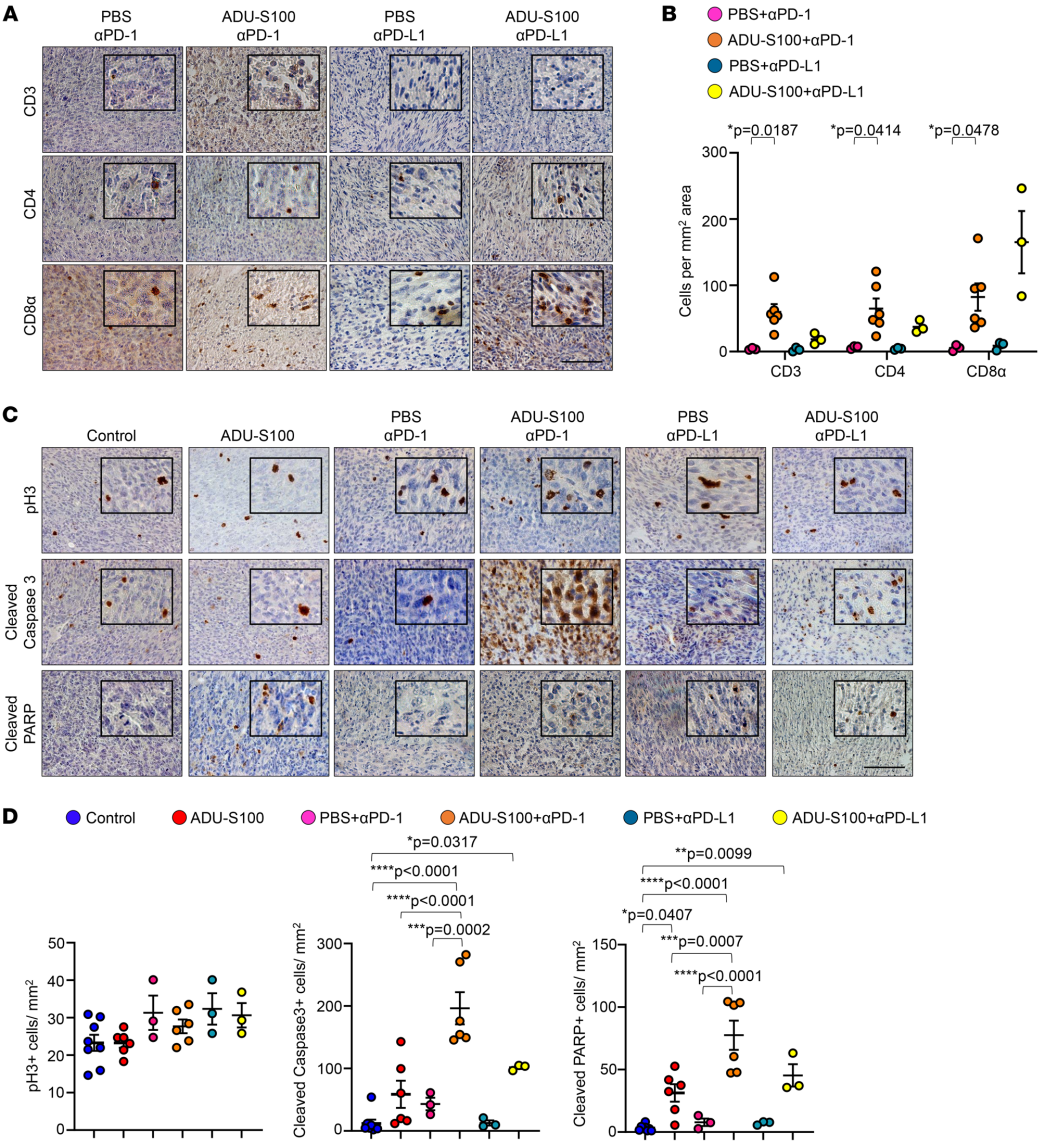
Combination treatment of *cisNP* mice with STING agonist plus ICB increases the expression of apoptotic markers in MPNSTs. (**A**) Paraffin sections from MPNSTs harvested from mice treated as indicated were stained for T cell markers. (**B**) Quantification of images in **A**. (**C**) Paraffin sections from MPNSTs harvested from mice treated as indicated were stained for p-H3, cleaved caspase 3, and cleaved PARP. (**D**) Quantification of p-H3^+^ cells, cleaved caspase 3^+^ cells, and cleaved PARP^+^ cells in tumors treated as indicated in **C**. Control, *n* = 8; ADU-S100, ADU-S100 plus anti–PD-1, *n* = 6; PBS plus anti–PD-1, PBS plus anti–PD-L1, ADU-S100 plus anti–PD-L1, *n* = 3. Data are presented as the mean ± SEM. *P < 0.05, **P < 0.01, ***P < 0.001, and ****P < 0.0001, by 2-tailed *t* test (**B**) and Tukey’s multiple-comparison test (**D**). Scale bars: 50 μm. Original magnification, ×80 (enlarged insets in **A** and **C**). See Methods for a detailed description of the staining methodology.

Our finding that STING agonist treatment could stimulate infiltration of PD-1–expressing T cells into tumors in our MPNST model indicated that these tumors might now be responsive to ICB. We therefore tested whether ICB treatment, together with a STING agonist, would have an inhibitory effect on tumor growth. Figure 4, A and B, shows the treatment arms and the combination treatment protocol, respectively, for the treatment of *cisNP* mice with the STING agonist ADU-S100 plus anti–PD-1 or anti–PD-L1 antibody. On day 12 following the start of treatment, tumors were measured and the mice euthanized. Similarly, we treated conditional MPNSTs with the combination of STING agonist and ICB (Supplemental Figure 5A). In contrast to the *cisNP* mice, conditional MPNST-bearing mice were kept alive until tumor volumes reached maximum limits or became ulcerated (in accordance with animal welfare guidelines). As a control, we also treated MPNST allografts in nude mice with the same combination treatment (Figure 4C). We found that tumors in *cisNP* mice treated with the combination of a STING agonist plus ICB were smaller compared with vehicle-treated mouse tumors (Figure 4, D and E, and Supplemental Figure 6, A–D), but we found no significant difference in tumor size in the allograft nude mice treated with vehicle versus those treated with the drug (Figure 4, D and E). Conditional MPNSTs that received the STING agonist plus ICB were significantly smaller than the controls (Supplemental Figure 5, B and C), and the mice lived longer than the control mice. This observation suggests that the MPNST growth delay initiated by STING activation was mediated through T cells. Indeed, IHC analysis of the tumors with a panel of T cell markers showed an increased presence of T cells in the tumors that received drug treatment (Figure 5, A and B, and Supplemental Figure 7A).

To further investigate the molecular mechanisms responsible for the tumor growth delay observed upon STING activation and ICB, we performed IHC for markers of cell proliferation and apoptosis. Cell proliferation as measured by phospho–histone H3 (p-H3) levels showed no significant difference among treatment groups (Figure 5, C and D). However, cell death, as measured by expression of the apoptosis markers cleaved caspase 3 and cleaved PARP was significantly increased in tumors treated with ADU-S100 alone and with ADU-S100 plus ICB (Figure 5, C and D, and Supplemental Figure 7B). Interestingly, MPNSTs that received the combination treatment of ADU-S100 plus anti–PD-1 showed significantly increased apoptotic cell death compared with those treated with ADU-S100 alone (Figure 5, C and D, and Supplemental Figure 7B). This, together with the observation that ADU-S100 plus anti–PD-1 tumors were significantly smaller than tumors treated with anti–PD-1 alone (Figure 4, D and E, and Supplemental Figure 5, B and C), suggests that ICB further facilitated the MPNST growth delay induced by STING activation.

We also wanted to investigate whether adding another checkpoint-blocking antibody in addition to anti–PD-1 and anti–PD-L1 would result in a further reduction of tumor volume. For this, we treated *cisNP* mice with anti–PD-1 and anti–CTLA-4 monoclonal antibodies on days 1 and 4, in combination with ADU-S100 (Supplemental Figure 8A). As a control, another set of *cisNP* mice received only ADU-S100 and anti–CTLA-4 (Supplemental Figure 8A). After 12 days, neither combination of ADU-S100 plus anti–PD-1 and anti–CTLA-4 nor ADU-S100 plus anti–CTLA-4 treatments showed a significant difference in tumor volume compared with ADU-S100 alone or ADU-S100 plus anti–PD-1 treatments (Supplemental Figure 8, B and C). Upon further investigation, we observed that CTLA-4 protein levels were not markedly different between the control- and ADU-S100–treated *cis*MPNSTs, which may explain why CTLA-4 inhibition did not challenge *cis*MPNST growth (Supplemental Figure 8D).

### STING activation and ICB accelerate complete regression of xenograft human MPNSTs.

**Figure 6:**
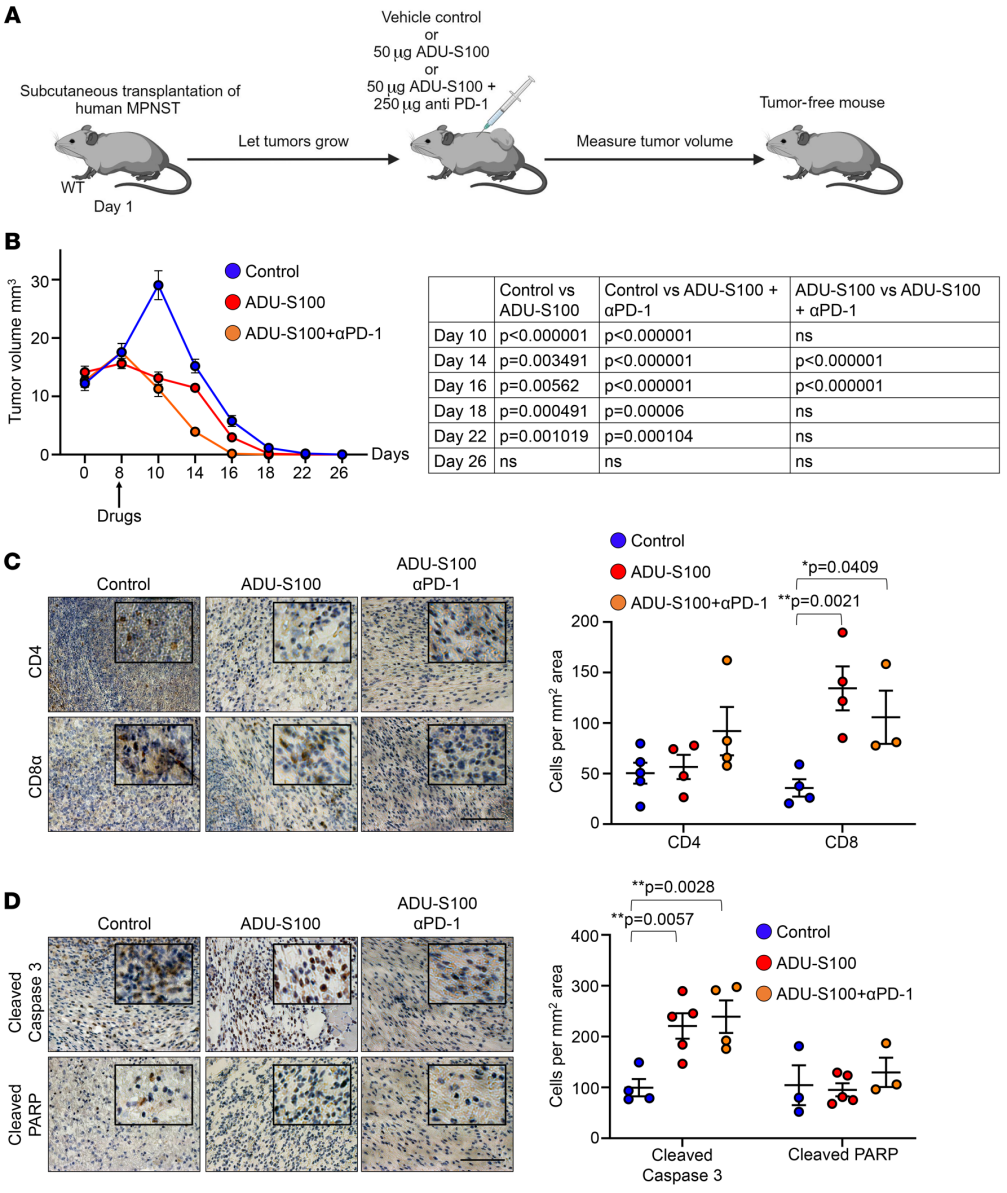
Combination treatment of xenograft human MPNST with a STING agonist plus ICB accelerates complete tumor regression. (**A**) Schema showing the design of the mouse xenograft MPNST model and the treatment regimen of ADU-S100 with ICB. (**B**) Change in xenograft MPNST volumes following the indicated treatments. Control, ADU-S100, and ADU-S100 plus anti–PD-1 (*n* = 15 each). (**C**) Paraffin sections from xenograft MPNSTs treated as indicated were stained for T cell markers. (**D**) Paraffin sections from xenograft MPNSTs treated as indicated were stained for cleaved caspase 3 and cleaved PARP. Control, *n* = 3–4; ADU-S100, *n* = 3–4; ADU-S100 plus anti–PD-1, *n* = 3–4. Data are presented as the mean ± SEM. *P < 0.05 and **P < 0.01, by Tukey’s multiple-comparison test. Scale bars: 50 μm. Original magnification, ×80 (enlarged insets in **C** and **D**).

Although we observed mouse MPNST growth delay in response to STING activation alone or following the combination treatment with ICB, their effect on human MPNSTs is not known. Therefore, to investigate the possibility of achieving complete human tumor regression, we treated a xenograft MPNST mouse model with either ADU-S100 or ADU-S100 plus anti–PD-1 (Figure 6A). WT immunocompetent mice will eventually reject the implanted human tissue. Indeed, as expected, subcutaneously transplanted human MPNSTs were eliminated within 1 month with vehicle treatment (Figure 6B). We reasoned that treatment with the STING agonist together with ICB might hasten this rejection and might do so faster than with STING agonist treatment alone. Interestingly, although vehicle-treated tumors continued to grow for a short period after implantation, tumors treated with ADU-S100 only or with ADU-S100 plus anti–PD-1 started shrinking soon after treatment (Figure 6B). We found that tumors treated with ADU-S100 only or with the combination treatment maintained a significantly smaller volume compared with the vehicle-treated tumors. Importantly, by day 14, tumors that received the combination treatment were significantly smaller than the tumors treated with ADU-S100 alone and completely regressed more quickly than their ADU-S100–treated counterparts (Figure 6B, and Supplemental Figure 9, A and B). As expected, xenograft tumors treated with ADU-S100 alone or ADU-S100 plus ICB showed significantly higher T cell infiltration and apoptotic cell death (Figure 6, C and D). However, the same human MPNST fragments transplanted into nude mice continued to grow despite the drug treatments, and unlike in immunocompetent mice, the tumors in the control group were not rejected by immunodeficient nude mice, suggesting a requirement for T cells to mediate the antitumor effects observed (Supplemental Figure 9C). Furthermore, cell death marked by cleaved caspase 3 and cleaved PARP expression was not significantly different among mice in the treatment groups but was significantly lower than that observed in WT mice in the respective treatment groups (Supplemental Figure 9, D and E).

Our data demonstrate that STING activation followed by ICB was more effective than ADU-S100 treatment alone at eliminating MPNSTs in vivo. These proof-of-principle experiments support the clinical testing of this combination treatment in patients with inoperable MPNSTs.

## Discussion

There are currently no effective drugs available for the treatment of MPNSTs, aggressive tumors that are the leading cause of death for patients with NF1. ICB has revolutionized cancer treatment, offering a durable response for tumors that are immune inflamed; however, many tumor types, including MPNSTs, are cold tumors lacking immune cell infiltration and thus are not good candidates for ICB therapy. To overcome this problem, various strategies to convert cold tumors to hot are currently being explored. These strategies include using low doses of radiation or oncolytic viruses and harnessing the innate immune system.

A couple of reports describing the use of viral treatments to boost immune infiltration in MPNSTs have recently been published. In 2021, Ghonime et al. reported that a multimodal oncolytic virus engineered to express EphrinA2, an antigen expressed by a variety of tumor types, is able to induce a robust immune therapeutic response in immune-competent mouse models of glioma and MPNST (42). A more recent report by Yan et al. demonstrated that viral treatment of MPNSTs has the ability to transform the immune desert environment using intratumoral delivery of inactivated modified vaccinia virus Ankara (MVA) to enhance immune infiltration into MPNSTs, making them amenable to ICB (43).

In this study, we harnessed the power of the innate immune system to facilitate immune destruction of murine MPNSTs. We found that STING agonist treatment of MPNSTs caused activation of the STING pathway, upregulation of cytokines and chemokines, and infiltration of immune cells, including T cells, into the tumor. Of note, we found that the STING agonist alone was able to significantly slow MPNST growth in our *cisNP* mice (percentage increase in tumor volume for control vs. ADU-S100 treatment, *P* = 0.0123; Figure 4, D and E); percentage increase in tumor volume for control vs. SA3 treatment, *P* = 0.0384; Supplemental Figure 2F). However, STING activation alone could not completely ablate the tumor. This is a phenomenon reported by others, who have used STING activation for tumor inflammation as well (26). In the case of MPNSTs, this could be due to immune escape of the tumor as a result of PD-L1 expression by a subset of tumor cells and its interaction with PD-1 on immune cells. Consequently, we observed enhanced tumor growth delay and significantly increased apoptosis upon STING activation followed by ICB compared with STING activation alone (for cleaved caspase 3: ADU-S100 vs. ADU-S100 plus anti–PD-1, *P* < 0.0001; for cleaved PARP: ADU-S100 vs. ADU-S100 plus anti–PD-1, *P* = 0.0007). Nevertheless, our experiments with ADU-S100 and ADU-S100 plus ICB had to be concluded within 12 days: *cisNP* mice formed aggressive MPNSTs that grew rapidly, and control animals (treated with vehicle only) had to be sacrificed because of the size of their tumors. Hence, we could neither follow the drug-treated tumors long enough to assess for complete regression nor determine the survival curves for these studies.

To circumvent this issue, we generated a xenograft MPNST model in which we subcutaneously transplanted human MPNSTs into immunocompetent WT mice. As we could carefully measure tumor growth, this provided a more manageable system to test our treatment regimens. Given our data from *cisNP* mice showing that STING agonist treatment (ADU-S100) followed by systemic PD-1 inhibition yielded the most beneficial results against MPNST growth, we tested the efficacy of ADU-S100 alone versus ADU-S100 plus anti–PD-1 in this xenograft mouse model. As expected with an immunocompetent host, the animals that received vehicle treatment only showed complete elimination of the xenograft tumor over time due to rejection of the human tissue by the murine immune system. However, STING activation alone enhanced this immune destruction, whereas STING activation followed by ICB further significantly accelerated it.

In this study, the STING agonist ADU-S100 was administered by intratumoral injection. Human MPNSTs are often located internally, with close proximity to complex nerve networks. Therefore, we used STING agonist 3 (SA3), administered intraperitoneally, to assess the efficiency of a systemically delivered drug in activating the STING pathway. SA3 showed promise by upregulating STING signaling, increasing T cell infiltration, and inhibiting MPNST growth in *cisNP* mice. Moreover, human primary MPNSTs tend to metastasize. Therefore, exploring whether STING activation and ICB can prime the immune system to target metastatic lesions is of importance in extending these studies to a clinical setting.

We have shown that STING activation reprograms the TME to enhance T cell infiltration into MPNSTs and sensitize them to ICB. It is worth discussing how benign pNFs might respond to STING activation compared with MPNSTs. T cells have been shown to be present in pNFs and play a positive role in pNF development. Since a pNF is a hamartoma with minimal genetic mutation compared with an MPNST, T cells probably recognize it as “self” (44, 45). However, MPNSTs, which have more neoantigens, resemble “non-self” to T cells that infiltrate following STING activation and therefore can be targeted for immune destruction (46). Thus, it is possible that STING activation, while restraining MPNST growth, might promote pNF progression by increasing T cell infiltration.

Combination treatment with STING agonist plus immune checkpoint inhibitors is currently being tested in clinical trials for some cancers. ADU-S100 was evaluated in a phase I clinical trial involving 47 patients with advanced/metastatic solid tumors or lymphomas, either alone or in combination with ipilimumab, an immune checkpoint inhibitor that targets CTLA-4 (NCT02675439). However, the trial was terminated due to lack of antitumor activity. Another phase I clinical trial testing the safety and maximum tolerated dose of the STING agonist TAK-500, alone or with pembrolizumab, a monoclonal antibody targeting PD-1, is currently recruiting patients with locally advanced or metastatic solid tumors, however, MPNST is not one of the eligible tumor types (ClinicalTrials.gov ID, NCT05070247). Our preclinical data support the testing of a STING agonist together with immune checkpoint inhibition in clinical trials for the treatment of inoperable MPNSTs.

## Methods

### Sex as a biological variable

Our study examined male and female humans and mice, and similar findings are reported for both sexes.

### Mice

Mouse colonies were maintained in a barrier facility at UT Southwestern. Mice were housed in standard cages that contained 3–5 mice per cage, with water and standard diet ad libitum and a 12-hour light/12-hour dark cycle. The *cisNP* mouse model has been previously described (27, 28).

### Genotyping

To determine the genotypes of the genetically modified mice, a 1 mm piece of tail was clipped from pups less than 2 weeks of age. Genomic DNA was extracted from this piece by incubation for 1.5 hours at 95°C in 50 mM NaOH. DNA lysates were then neutralized at room temperature with 1 M Tris-HCl (pH 7). To genotype *cisNP* mice, the following primers were used for the *Nf1* allele: 5940 (5′-GTATTGAATTGAAGCACCTTTGTTTGG-3′), 5941 (5′-GCGTGTTCGAATTCGCCAATG-3′), and 5942 (5′-CTGCCCAAGGCTCCCCCAG-3′), generating a 194 bp band for the WT and a 340 bp band for the heterozygote. For the *Tp53* allele, the GT-P53-1 (5′-TATACTCAGAGCCGGCCT-3′), GT-P53-2 (5′-CATTCAGGACATAGCGTTGG-3′), and GT-P53-3 (5′-ACAGCGTGGTGGTACCTTAT-3′) primers were used, generating a 430 bp band for the WT and a 650 bp band for the heterozygote. To genotype the conditional MPNST mice, the following primers were used for the *Nf1^fl/fl^* allele: 15228 (5′-ACCTCTCTAGCCTCAGGAATGA-3′), 15229 (5′-CTTCAGACTGATTGTTGTACCTGA-3′), and 15588 (5′-TGATTCCCACTTTGTGGTTCTAAG-3′), generating a 480 bp band for the WT and a 350 bp band for the mutant. For the *p53^fl/fl^* allele, the primers P53-i5F (5′-GGGGAGTTGTCTTTCGTGTGA-3′), P53-i6F (5′-TGTGCCGAACAGGTGGAATA-3′), and P53-i7R (5′-CTAACCTACCACGCGCCTTC-3′) were used, generating a 275 bp band for the WT and a 314 bp band for the mutant. For the *PLP-CreERT2* allele, the primers PLP-Ex2F (5′-CCTCGTATGCGTACCTGACT-3′), Cre-R69 (5′-TGTGCCGAACAGGTGGAAT-3′), and PLP-In3R (5′-CATTAGACCGCTACCTGCCA-3′) were used, generating a 526 bp band for the WT and a 190 bp band for the mutant. The DNA sequences were amplified with 2XTaq RED Master Mix (Apex).

### MPNST allografts and xenografts in mice

#### Allografts.

Athymic nude mice were purchased from Charles River Laboratories (stock no. 553). They were subcutaneously injected with 5 million *cis*MPNST cells per injection site. Once the injected cells formed tumors measuring 5 mm in diameter, the mice were treated with a STING agonist(s) and/or monoclonal anti–PD-1/anti–PD-L1 antibodies as described below.

#### Xenografts.

Surgically resected metastatic MPNST tissue was subcutaneously transplanted into nude mice to generate a xenograft model. Samples of these tumors were then transplanted subcutaneously into WT mice to generate xenograft MPNSTs in immunocompetent mice.

### Cell culturing

H358 cells (shared by the Kate O’Donnell laboratory at UT Southwestern) were KRAS mutant human lung cancer cells that expressed PD-L1 (47). S462 cells were human NF1–related MPNST cells (SCC414, MilliporeSigma). The *cis*MPNST cell line was generated in the laboratory by harvesting tumors from *cis*MPNST mice. The HTS-Luc MPNST cell line was described before (48). Human and mouse MPNST cell lines were cultured in DMEM high-glucose (R8756, MilliporeSigma) supplemented with 10% FBS (5628, MilliporeSigma), 1% sodium pyruvate (S8636, MilliporeSigma), GlutaMAX (35050079, Gibco, Thermo Fisher Scientific) and 1% penicillin/streptomycin (T4049). For STING agonist treatments, cells were seeded in 6-well plates at a 3 × 10^5^ cells/well concentration and allowed to grow to 80% confluence. The cells were then treated with 10 μm ADU-S100 for 8, 18, 24, or 48 hours. Cells were then washed with PBS and harvested for qRT-PCR and immunoblotting.

### Real-time and qRT-PCR

Tumor tissue samples were frozen in liquid nitrogen and pulverized while cold. Total RNA from these tissues and cultured MPNST cells were extracted using TRI Reagent (T9424, Thermo Fisher Scientific), and 1 μg RNA was reverse transcribed with an iScript Select cDNA Synthesis Kit (1708897, Bio-Rad). The primer sequences are listed in Supplemental Table 1. qRT-PCR reaction mixtures were prepared with iTaq Universal SYBR Green Supermix (172-5124, Bio-Rad), and reactions were performed using the QuantStudio 3 Real-Time PCR System (Thermo Fisher Scientific). Ct values were normalized to the housekeeping gene *Gusb*.

### Immunoblotting

Protein lysates from tumor tissue and cultured tumor cells were made using RIPA buffer (8990, Thermo Fisher Scientific) containing protease and phosphatase inhibitors (88265 and A32959, respectively, Thermo Fisher Scientific). The following antibodies were used for immunoblotting: STING (13647S, Cell Signaling Technology), TBK1 (3504T, Cell Signaling Technology), p-TBK1 (s172) (5483T, Cell Signaling Technology), IRF3 (MA5-32348, Invitrogen, Thermo Fisher Scientific), p-IRF3 (4947S, Cell Signaling Technology), NF-κB (ab16502, Abcam), p–NF-κB (3033S, Cell Signaling Technology), PD-L1 (ab213480, Abcam, 66248-1-Ig, Proteintech), GAPDH (SC-32233, Santa Cruz Biotechnology), vinculin (4650S, Cell Signaling Technology), Foxp3 (ab215206, Abcam), and CTLA-4 (BE0164, Bio X Cell).

### IHC and immunofluorescence

Tumors were fixed in formalin for at least 48 hours and then processed and embedded in paraffin blocks using a Citadel 2000 Wax Bath. Serial 5 μm sections were prepared for IHC and immunofluorescence (IF). Paraffin sections were deparaffinized in xylene and rehydrated using ethanol and water. Antigens were retrieved using citrate antigen retrieval buffer (pH 6.0) or TE buffer (pH 9.0). Sections were then blocked and incubated with primary and secondary antibodies using standard methods. The following antibodies were used for IHC: CD20 (BS-0080R, Bioss), CD3 (ab16669, Abcam), CD4 (ab183685, Abcam), CD8α (PA5-81344), Iba1 (019-19741, Thermo Fisher Scientific), iNOS (ab15323, Abcam), mannose receptor (ab64693, Abcam), p-H3 (ser10) (9701s, Cell Signaling Technology), cleaved caspase 3 (9661S, Cell Signaling Technology), and cleaved PARP (9488S, Cell Signaling Technology). For IF, paraffin sections were incubated with anti-CD3 (ab16669, Abcam) and anti–PD-1 (ab214421, Abcam; 66220-1-Ig, Proteintech) antibodies overnight at room temperature followed by incubation with goat anti–rabbit IgG-Cy3 secondary antibody (Jackson ImmunoResearch). After staining, images were acquired with an Olympus IX73 microscope. For quantification, 3–5 different fields of each sample were imaged. These fields were selected randomly, avoiding the tumor borders. The target cell type was counted using the ImageJ Cell Counter extension (NIH), and the average number of cells per square millimeter was calculated. It should be noted that in the control treatments, some of the markers that were assessed were scarcely expressed. In this situation, the development reaction in IHC was continued for a longer period to capture a positive signal. However, this resulted in higher background staining. At image acquisition, the same parameters were maintained for all tumor samples, regardless of the treatment, resulting in some of the images from “control” sections having higher background staining (e.g., Figure 3A, PD-1). Similar high background staining was also observed in the control sections in the IF images (e.g., Figure 3F), as CD3^+^ and PD-1^+^ cells were scarce in those sections.

### STING agonist treatment and ICB

When *cisNP* mice or conditional MPNST-bearing mice or athymic mice with *cis*MPNST allografts developed a tumor with at least 1 dimension reaching 5 mm, they were treated with intratumoral injections of 50 μg of the STING agonist ADU-S100 (HY-12885B, MedChemExpress). The day of the first injection was considered day 1, and the injections were repeated on days 4 and 7. Each day the tumor or tumors were measured at 3 perpendicular planes designated as the length (L), width (W), and depth (D), and the tumor volume was calculated as (L × W × D)/2. On day 12, mice were sacrificed, and the tumors were harvested (Figure 2A, Figure 4, B and C, and Supplemental Figure 5A). For the welfare of the mice, the tumors were not allowed to grow to more than 2 cm in diameter. Therefore, the length of the experiments was determined from results of pilot studies showing how long it took vehicle-treated tumors to reach 2 cm in diameter. For the human xenograft tumor studies, 50 μg ADU-S100 or 50 μg ADU-S100 with 250 μg mouse monoclonal anti–PD-1 antibody (BE0146, Bio X Cell) were injected intratumorally on day 8. Alternatively, mice that were treated with the STING agonist SA3 (HY-103665, MedChemExpress) received a single intraperitoneal injection of 50 mg/kg BW and were sacrificed on day 12. To combine STING activation with ICB, intraperitoneal injections of 250 μg mouse monoclonal anti–PD-1 antibody (BE0146, Bio X Cell) or 100 μg mouse monoclonal anti–PD-L1 antibody (BE0146, Bio X Cell) or 300 μg mouse monoclonal anti–CTLA-4 antibody (BE0164, Bio X Cell) were given on days 1 and 4 in addition to STING agonists, and mice were euthanized on day 12. The dosing regimens are shown in Table 1.

### Statistics

All data points shown in the figures resulted from biological replicates. The number of replicates are described in the figure legends. Unless otherwise stated in the figure legends, a 2-tailed *t* test was used to determine statistical significance. A *P* value of less than 0.05 was considered statistically significant. Data are presented as the mean ± SEM or ± SD, as indicated in the figure legends.

### Study approval

The care and use of animals in this study were approved by the IACUC of the University of Texas Southwestern Medical Center. The use of deidentified human tissue was approved by the IRB of the University of Texas Southwestern Medical Center.

### Data availability

All represented data are included in the Supporting Data Values file and will be available from the corresponding author upon request.

## Author contributions

BNS, RMM, and LQL conceptualized the study. BNS, SC, AS, LM, and ZC performed experiments. BNS and RMM wrote the original draft of the manuscript. All authors reviewed and edited the manuscript. LQL supervised the study and obtained funding.

## Supplementary Material

Supplemental data

Unedited blot and gel images

Supporting data values

## Figures and Tables

**Table 1:**
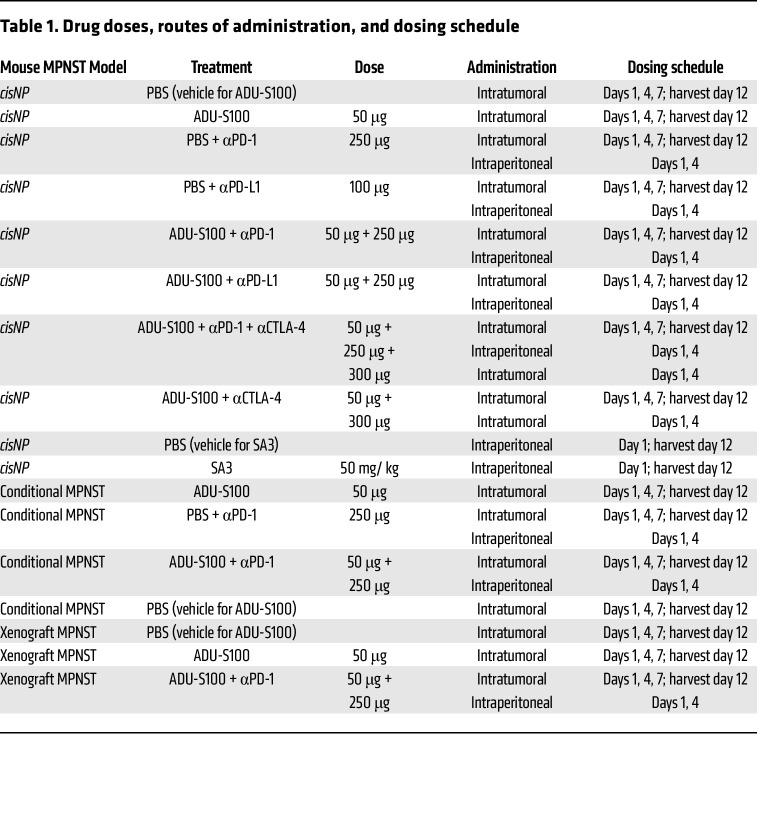
Drug doses, routes of administration, and dosing schedule

## Version 1 03/19/2024

In-Press Preview

## Version 2 05/15/2024

Electronic publication
