# Supplementary material for: STING activation reprograms the microenvironment to sensitize NF1-related malignant peripheral nerve sheath tumors for immunotherapy
Source: J Clin Invest. 2024 Mar 19;134(10):e176748. doi: 10.1172/JCI176748 (PMC11093615; doi:10.1172/JCI176748)
Supplement: Supplemental data [file jci-134-176748-s215.pdf]

Supplemental Figures and Table for:

STING Activation Reprograms the Microenvironment to Sensitize NF1-related Malignant Peripheral Nerve Sheath Tumors for Immunotherapy

Bandarigoda N. Somatilaka[1], Laasya Madana[1], Ali Sadek[1], Zhiguo Chen[1], Sanjay Chandrasekaran[2,3], Renee M. McKay[1], and Lu Q. Le[1,2,4,5,6,7]

[1]Department of Dermatology, [2] Simmons Comprehensive Cancer Center, [3]Department of Internal Medicine, Division of Hematology/Oncology, [4]UTSW Comprehensive Neurofibromatosis Clinic, [5]Hamon Center for Regenerative Science and Medicine, [6]O'Donnell Brain Institute, University of Texas Southwestern Medical Center at Dallas, Dallas, Texas, 75390-9069, USA, [7]Department of Dermatology, University of Virginia School of Medicine, Charlottesville, VA, 22903, USA

Author for correspondence:

Lu Q. Le, M.D., Ph.D.
Professor and Chair
Department of Dermatology
University of Virginia School of Medicine
Phone: (434) 982-5974
Fax: (434) 244-4504
E-mail: bkn6qd@uvahealth.org

Short title: Reprogramming MPNST for immune checkpoint blockade

Conflicts of interest: The authors have declared that they have no competing interests.

Keywords

Neurofibromatosis Type 1, NF1, neurofibroma, plexiform neurofibroma, malignant peripheral nerve sheath tumor, MPNST, STING, immune checkpoint blockade, ICB, ADU-S100

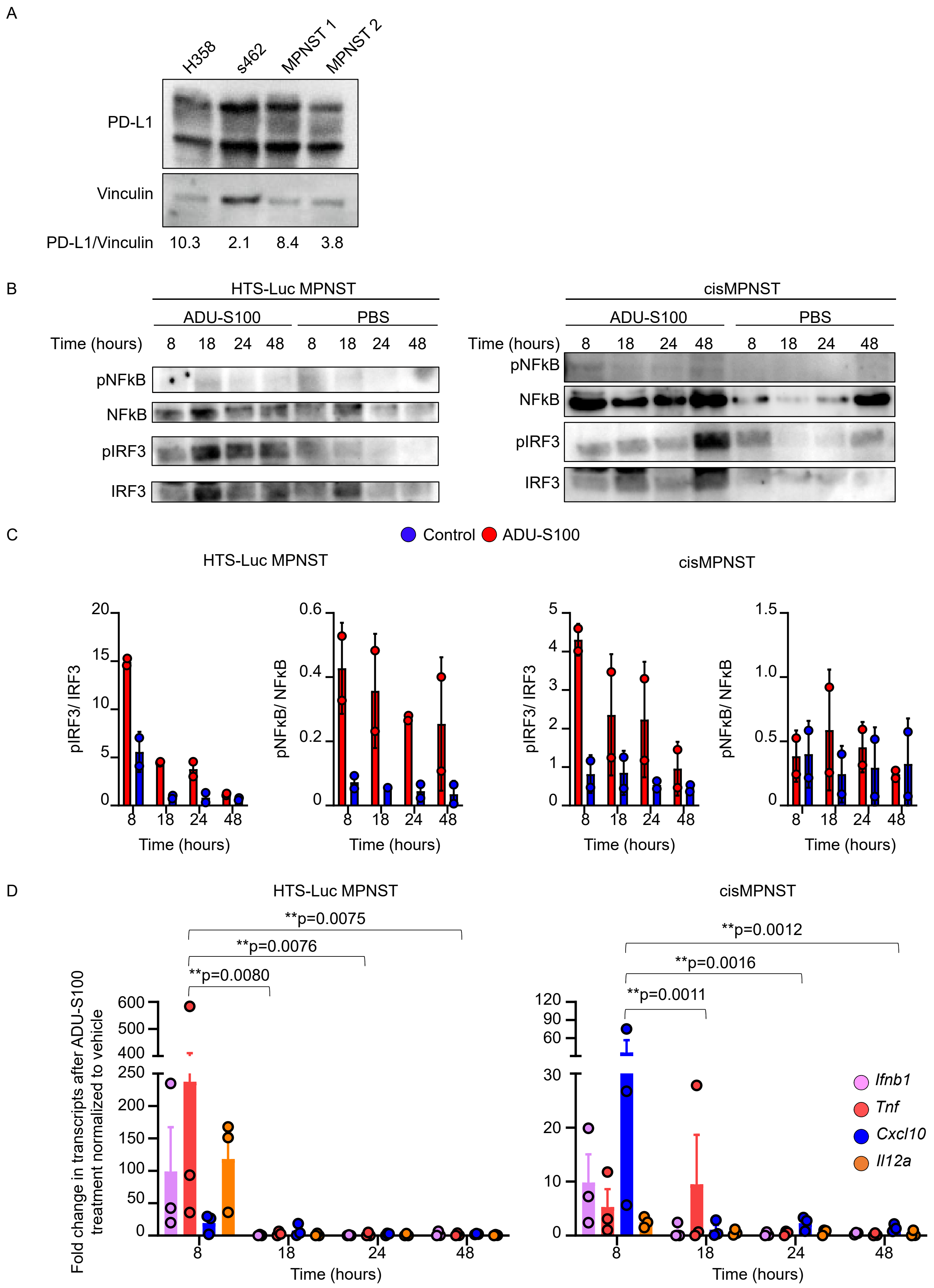

A
H358
s462
MPNST 1
MPNST 2
PD-L1
Vinculin
PD-L1/Vinculin 10.3 2.1 8.4 3.8
B
HTS-Luc MPNST
ADU-S100
PBS
Time (hours) 8 18 24 48 8 18 24 48
pNFκB
NFκB
pIRF3
IRF3
cisMPNST
ADU-S100
PBS
Time (hours) 8 18 24 48 8 18 24 48
pNFκB
NFκB
pIRF3
IRF3
C
Control
ADU-S100
HTS-Luc MPNST
cisMPNST
pIRF3/ IRF3
pNFκB/ NFκB
Time (hours)
D
HTS-Luc MPNST
**p=0.0075
**p=0.0076
**p=0.0080
Fold change in transcripts after ADU-S100 treatment normalized to vehicle
Time (hours)
cisMPNST
**p=0.0012
**p=0.0016
**p=0.0011
Ifnb1
Tnf
Cxcl10
Il12a

Supplemental Figure 1. STING agonist ADU-S100 activates the STING pathway in MPNST cell lines. (**A**) Western blot of the indicated cell lines and 2 cisMPNST tumors for PD-L1. The total PD-L1 band intensity normalized to that of vinculin is shown below each lane. (**B**) Western blot analysis for expression of the indicated proteins in mouse MPNST cell lines derived from *Nf1* and *p53* null skin progenitor cells (HTS-Luc MPNST) or harvested from *cisNP* mice (*cis*MPNST) treated with vehicle control (PBS) or ADU-S100. (**C**) Quantified protein band intensities from (**B**) are shown graphically. Data shown are as mean ± SD. (**D**) PCR analysis of fold change in cytokine gene expression (*Ifnb1*, *Tnf*, *Cxcl10*, and *Il12a*) in MPNST cells as in (**B**) treated with PBS (n=3/ time point) or ADU-S100 (n=3/ time point). Data are represented as mean ± SEM and p values are determined by Tukey's multiple comparisons test as indicated (D). $^{**}P < 0.01$.

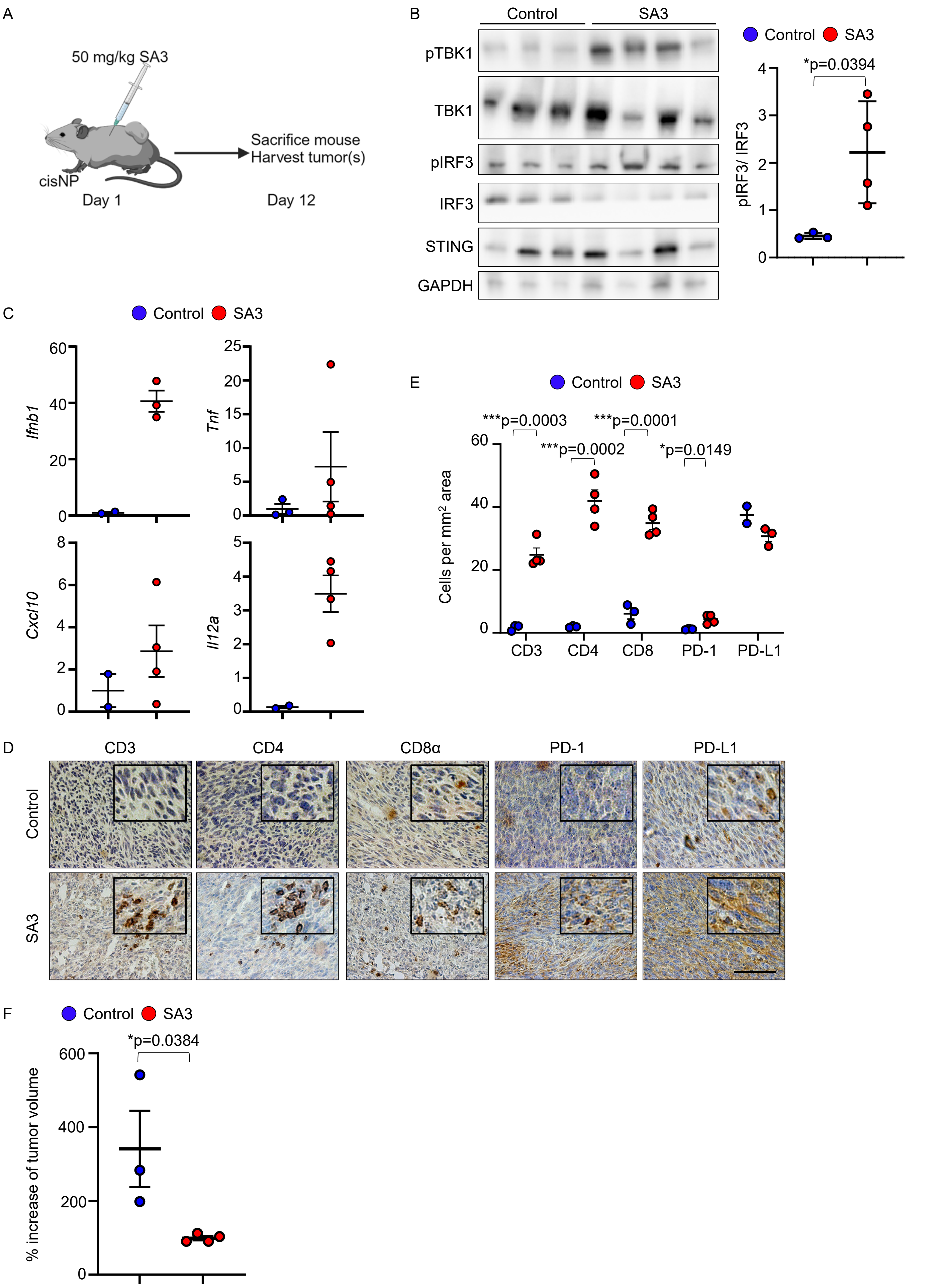
A
50 mg/kg SA3
cisNP
Sacrifice mouse
Harvest tumor(s)
Day 1
Day 12
B
Control
SA3
pTBK1
TBK1
pIRF3
IRF3
STING
GAPDH
Control SA3
*p=0.0394
pIRF3/ IRF3
0
1
2
3
4
C
Control SA3
Ifnb1
0
20
40
60
Tnf
0
5
10
15
20
25
Cxcl10
0
2
4
6
8
Il12a
0
1
2
3
4
5
E
Control SA3
***p=0.0003
***p=0.0002
***p=0.0001
*p=0.0149
Cells per mm² area
0
20
40
60
CD3
CD4
CD8
PD-1
PD-L1
D
CD3
CD4
CD8α
PD-1
PD-L1
Control
SA3
F
Control SA3
*p=0.0384
% increase of tumor volume
0
200
400
600

Supplemental Figure 2. SA3 treatment of *cisNP* mice activates the STING pathway in tumors. (**A**) Schema of SA3 treatment protocol. (**B**) Western blot analysis for expression of the indicated proteins in MPNSTs harvested from *cisNP* mice treated with vehicle control or SA3. Quantified protein band intensities for pIRF3/ IRF3 are shown graphically on the right. (**C**) PCR analysis of fold change in cytokine gene expression (*Ifnb1*, *Tnf*, *Cxcl10*, and *Il12a*) in *cis*MPNSTs harvested from control-treated (n=3) and SA3-treated (n=4) mice. (**D**) Paraffin sections of MPNSTs harvested from vehicle-treated or SA3 treated *cisNP* mice were stained with antibodies against CD3, CD4, CD8α, PD-1, and PD-L1 and quantified (**E**). (**F**) Percent increase in tumor volume was measured in control-treated or SA3-treated *cisNP* mice. Data are represented as mean ± SEM and p values are determined by two-tailed t test as indicated. *$P < 0.05$. Scale bar: 50 μm.

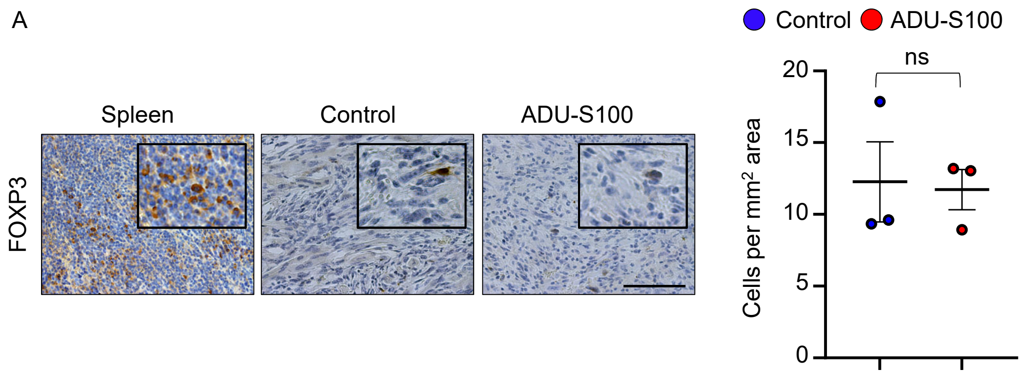
A
Control
ADU-S100
Spleen
Control
ADU-S100
FOXP3
ns
Cells per mm² area
20
15
10
5
0

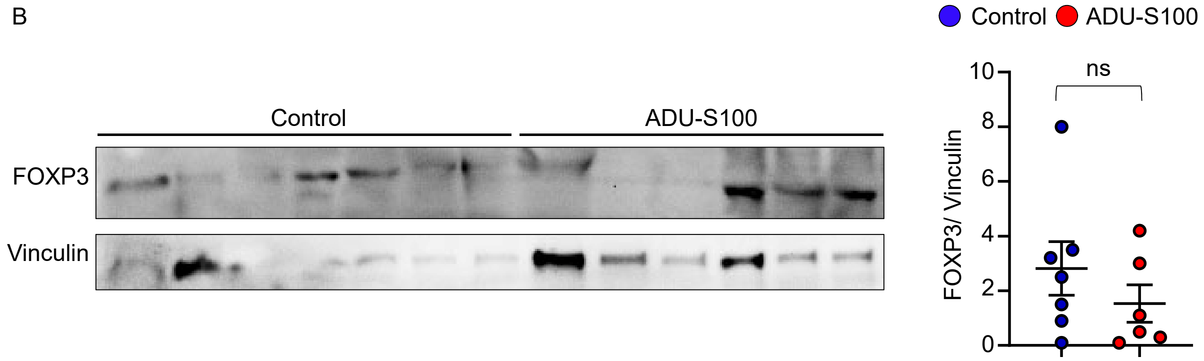
B
Control
ADU-S100
Control
ADU-S100
FOXP3
Vinculin
ns
FOXP3/ Vinculin
10
8
6
4
2
0

Supplemental Figure 3. *Foxp3* expression is unaltered upon ADU-S100 treatment. (**A**) Paraffin sections of murine spleen and control-treated (n=3) or ADU-S100-treated (n=3) *cis*MPNST are stained for FOXP3. Quantification of FOXP3-positive cells is shown on the right. (**B**) Western blot analysis for expression of FOXP3 in MPNSTs harvested from *cisNP* mice treated with vehicle control (n=7) or ADU-S100 (n=6). Quantified protein band intensities are shown graphically on the right. Data are represented as mean ± SEM and p values are determined by unpaired t test as indicated. ns = not significant.

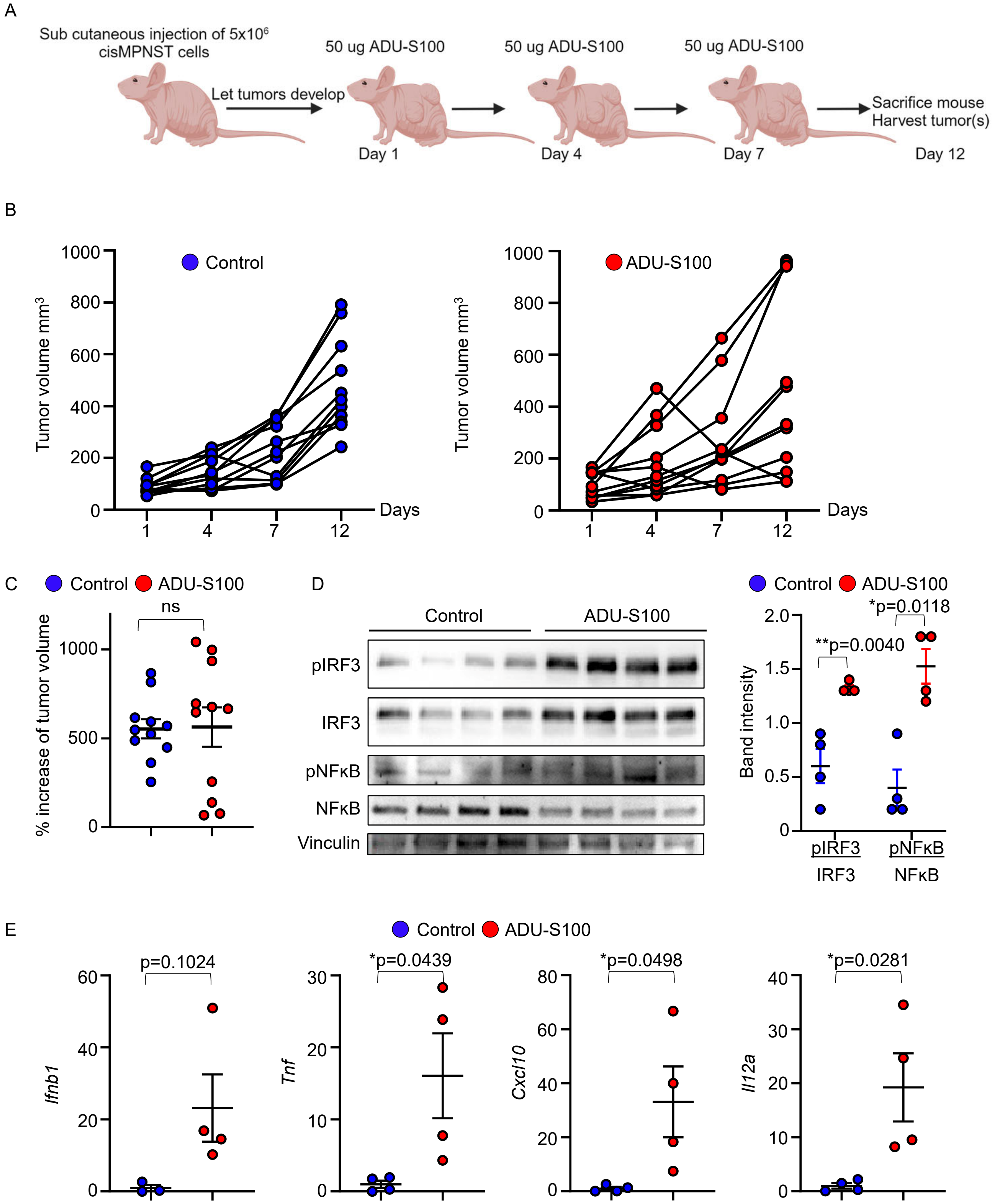
A
Sub cutaneous injection of 5x10⁶ cisMPNST cells
Let tumors develop
50 ug ADU-S100
50 ug ADU-S100
50 ug ADU-S100
Day 1
Day 4
Day 7
Sacrifice mouse
Harvest tumor(s)
Day 12
B
Control
ADU-S100
Tumor volume mm3
Days
C
Control ADU-S100
ns
% increase of tumor volume
D
Control
ADU-S100
pIRF3
IRF3
pNFκB
NFκB
Vinculin
Band intensity
**p=0.0040
*p=0.0118
pIRF3 / IRF3
pNFκB / NFκB
E
Control ADU-S100
p=0.1024
*p=0.0439
*p=0.0498
*p=0.0281
Ifnb1
Tnf
Cxcl10
Il12a

Supplemental Figure 4. STING agonist does not decrease MPNST volume in allograft MPNST in nude mice. (**A**) Schema of ADU-S100 treatment protocol in athymic nude mice bearing *cis*MPNST. (**B**) Tumor volume change over time in response to indicated treatments. (**C**) Tumor volume increase in athymic mice treated with PBS control (n=11) or ADU-S100 (n=11) as shown in (**A**). (**D**) Western blot analysis for expression of the indicated proteins in MPNSTs harvested from athymic mice treated with vehicle control (n=4) or ADU-S100 (n=4) for 24 hours. Quantified protein band intensities are shown graphically on the right. (**E**) PCR analysis of fold change in cytokine gene expression (*Ifnb1*, *Tnf*, *Cxcl10*, and *Il12a*) in *cis*MPNSTs harvested from control-treated (n=4) and ADU-S100-treated (n=4) athymic mice 24 hours after treatment. Data are represented as mean ± SEM and p values are determined by two-tailed t test with respect to vehicle control. $*P < 0.05$, $**P < 0.01$, ns = not significant.

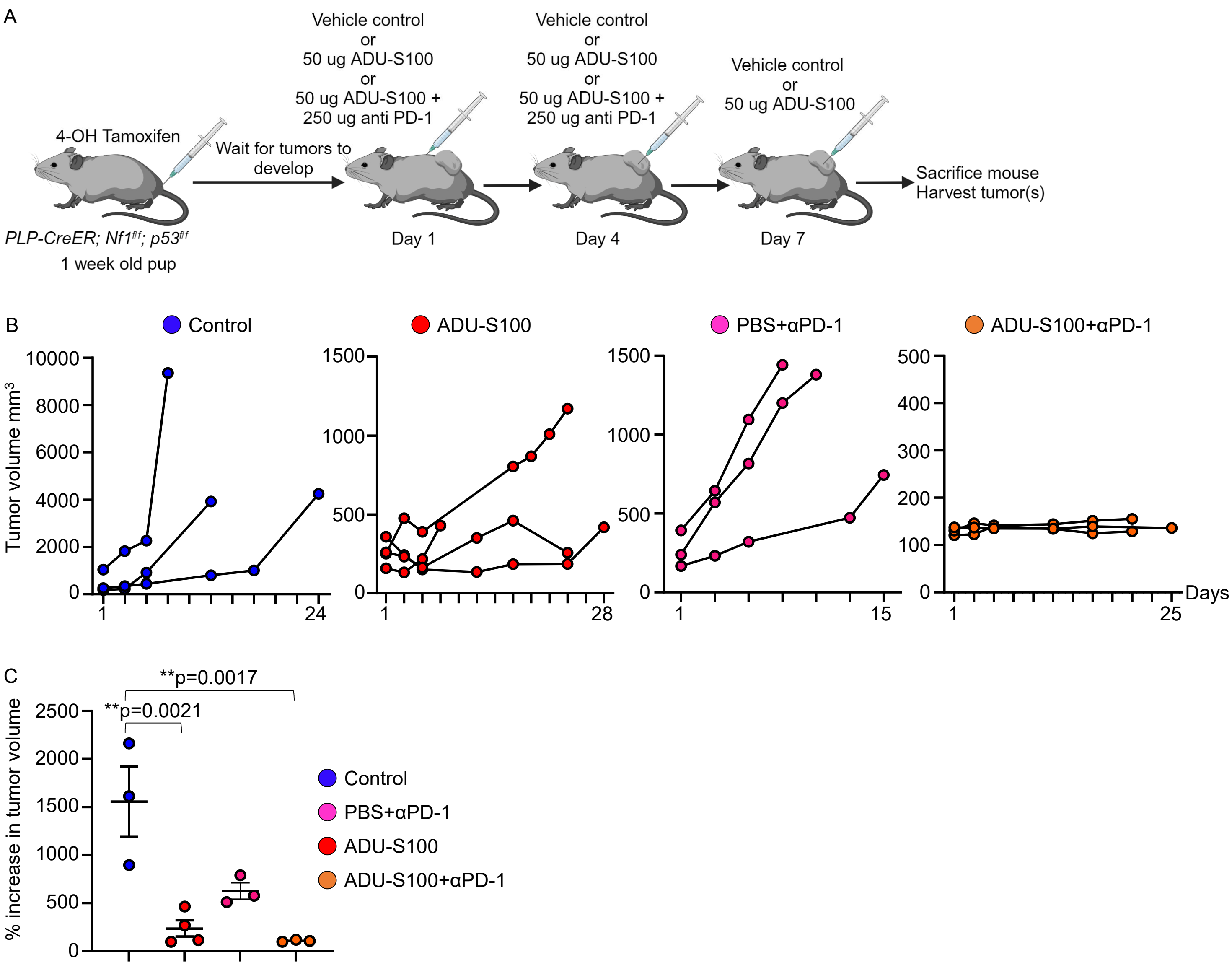
A
Vehicle control
or
50 ug ADU-S100
or
50 ug ADU-S100 +
250 ug anti PD-1
Vehicle control
or
50 ug ADU-S100
or
50 ug ADU-S100 +
250 ug anti PD-1
Vehicle control
or
50 ug ADU-S100
4-OH Tamoxifen
Wait for tumors to develop
Sacrifice mouse
Harvest tumor(s)
PLP-CreER; Nf1f/f; p53f/f
1 week old pup
Day 1
Day 4
Day 7
B
Control
ADU-S100
PBS+αPD-1
ADU-S100+αPD-1
Tumor volume mm3
Days
C
**p=0.0017
**p=0.0021
% increase in tumor volume
Control
PBS+αPD-1
ADU-S100
ADU-S100+αPD-1

Supplemental Figure 5. Combination treatment of STING agonist and ICB decreases MPNST growth in *PLP-Cre; Nf1$^{f/f}$; p53$^{f/f}$* mice. (**A**) Schema of STING activation and ICB combination treatment protocol in *PLP-Cre; Nf1$^{f/f}$; p53$^{f/f}$* mice. (**B**) Tumor volume change of MPNST in *PLP-Cre; Nf1$^{f/f}$; p53$^{f/f}$* mice in response to the indicated treatments. (**C**) Percent increase in tumor volume in *PLP-Cre; Nf1$^{f/f}$; p53$^{f/f}$* mice upon indicated treatments. Control, n=3; ADU-S100, n=4; PBS + αPD-1, n=3; ADU-S100 + αPD-1, n=3. Data are represented as mean ± SEM and p values are determined by Tukey's multiple comparisons test as indicated. *$P < 0.05$, **$P < 0.01$.

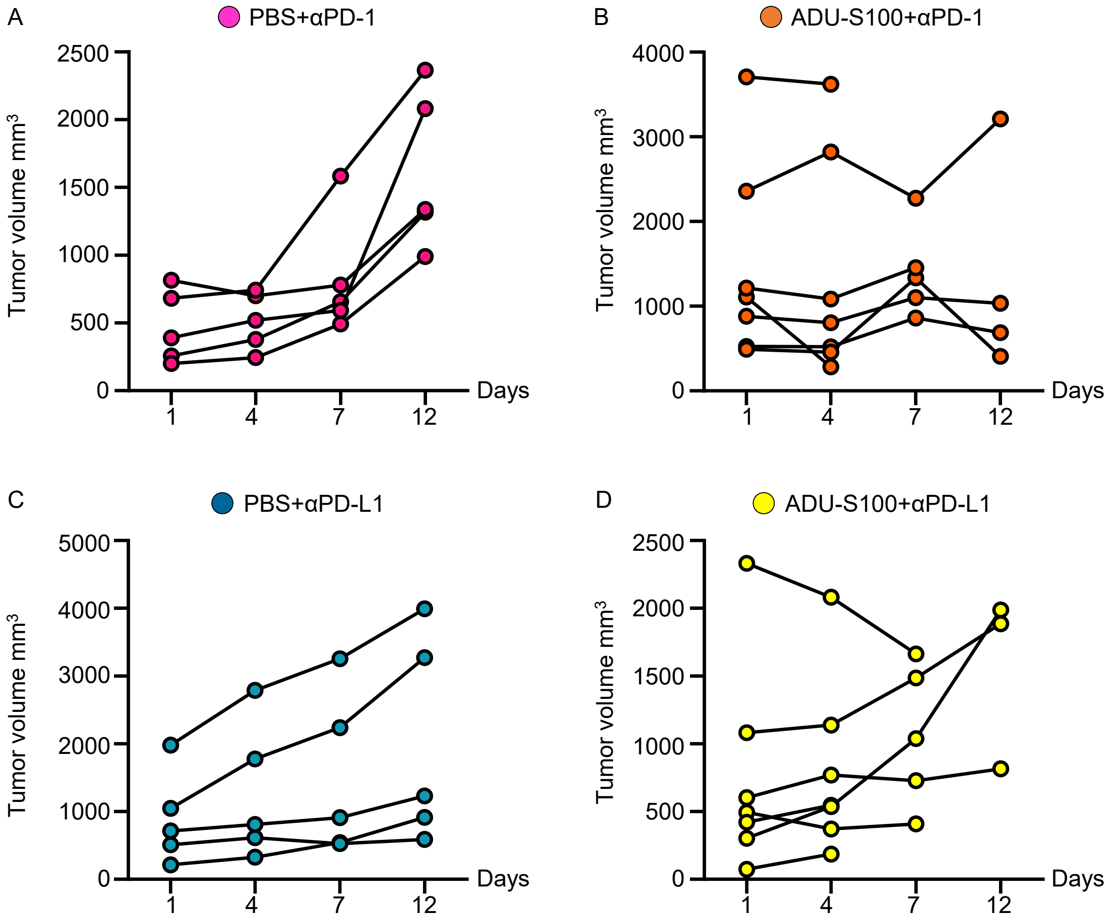
A
PBS+αPD-1
Tumor volume mm3
2500
2000
1500
1000
500
0
1
4
7
12
Days
B
ADU-S100+αPD-1
Tumor volume mm3
4000
3000
2000
1000
0
1
4
7
12
Days
C
PBS+αPD-L1
Tumor volume mm3
5000
4000
3000
2000
1000
0
1
4
7
12
Days
D
ADU-S100+αPD-L1
Tumor volume mm3
2500
2000
1500
1000
500
0
1
4
7
12
Days

Supplemental Figure 6. Combination treatment of *cis*MPNSTs with STING activation plus ICB slows tumor growth. Tumor volume change over time in *cisNP* mice treated with (**A**) PBS + αPD-1, (**B**) ADU-S100 + αPD-1, (**C**) PBS + αPD-L1, or (**D**) ADU-S100 + αPD-L1.

A

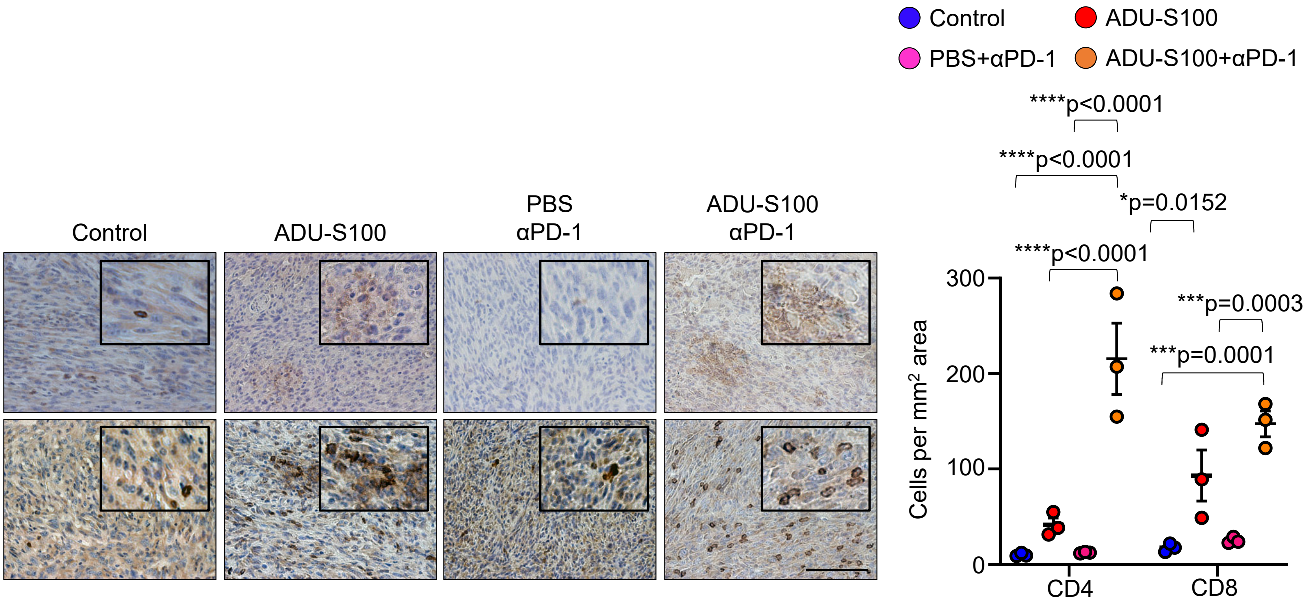
Control
ADU-S100
PBS+αPD-1
ADU-S100+αPD-1
Control
ADU-S100
PBS
αPD-1
ADU-S100
αPD-1
CD4
CD8α
****p<0.0001
****p<0.0001
*p=0.0152
****p<0.0001
***p=0.0003
***p=0.0001
Cells per mm² area
300
200
100
0
CD4
CD8

B

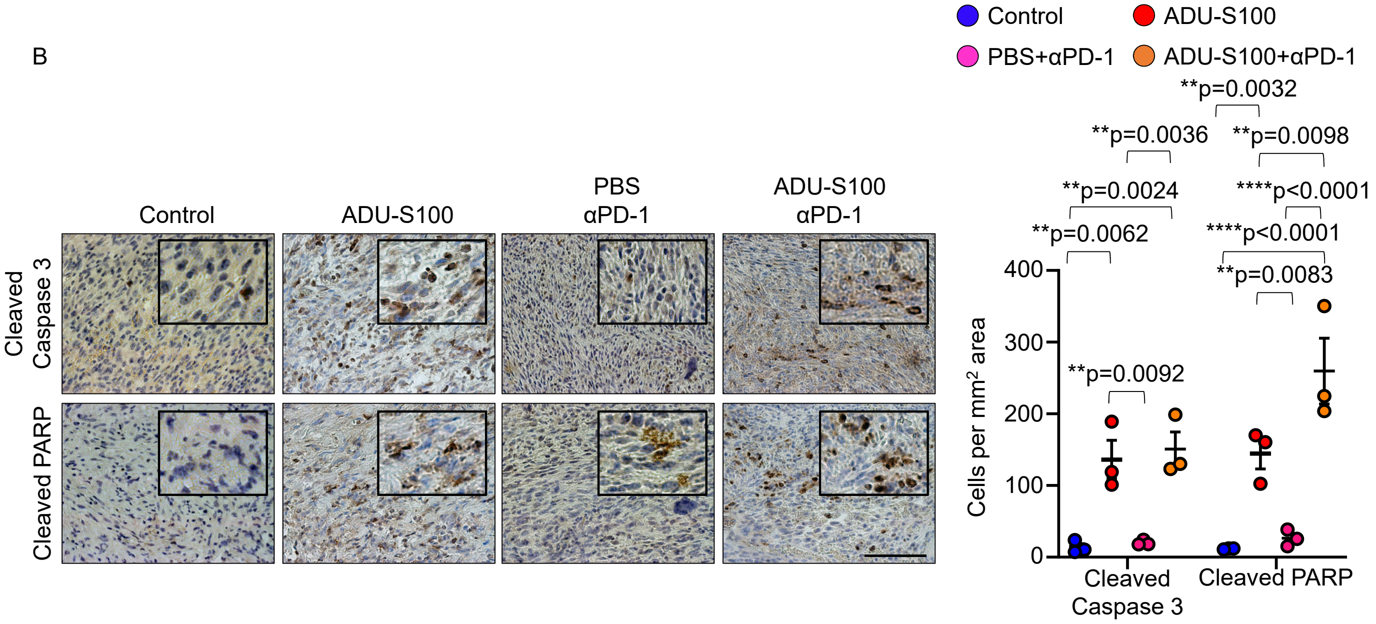
Control
ADU-S100
PBS+αPD-1
ADU-S100+αPD-1
Control
ADU-S100
PBS
αPD-1
ADU-S100
αPD-1
Cleaved
Caspase 3
Cleaved PARP
**p=0.0032
**p=0.0036
**p=0.0098
**p=0.0024
****p<0.0001
**p=0.0062
****p<0.0001
**p=0.0083
**p=0.0092
Cells per mm² area
400
300
200
100
0
Cleaved
Caspase 3
Cleaved PARP

Supplemental Figure 7. Combination treatment of STING agonist plus ICB increases T cell infiltration and expression of apoptotic markers in MPNST in *PLP-Cre; Nf1$^{f/f}$; p53$^{f/f}$* mice.

(**A**) Paraffin sections from MPNSTs harvested from *PLP-Cre; Nf1$^{f/f}$; p53$^{f/f}$* mice treated as indicated were stained for T cell markers. Quantification of images are shown on the right. (**B**) Paraffin sections from MPNSTs in (**A**) stained for Cleaved Caspase 3 and Cleaved PARP. Quantification of images are shown on the right. Control, n=3; ADU-S100, n=3; PBS + αPD-1, n=3; ADU-S100 + αPD-1, n=3. Data are represented as mean ± SEM and p values are determined by Tukey's multiple comparisons test as indicated. $^{*}P < 0.05$, $^{**}P < 0.01$, $^{***}P < 0.001$. Scale bar: 50 μm.

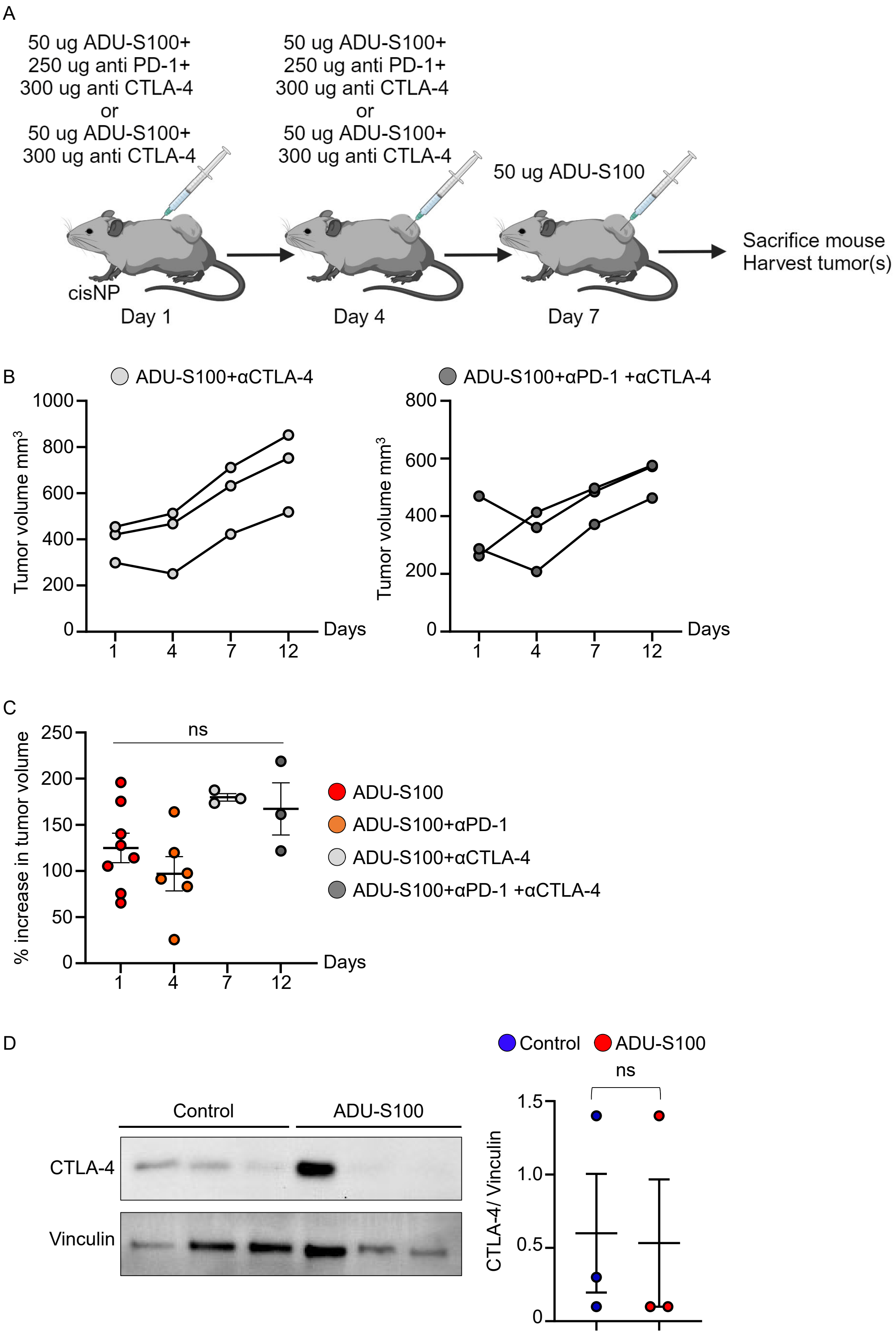
A
50 ug ADU-S100+
250 ug anti PD-1+
300 ug anti CTLA-4
or
50 ug ADU-S100+
300 ug anti CTLA-4
50 ug ADU-S100
Sacrifice mouse
Harvest tumor(s)
cisNP
Day 1
Day 4
Day 7
B
ADU-S100+αCTLA-4
ADU-S100+αPD-1 +αCTLA-4
Tumor volume mm3
Days
C
% increase in tumor volume
ns
ADU-S100
ADU-S100+αPD-1
ADU-S100+αCTLA-4
ADU-S100+αPD-1 +αCTLA-4
Days
D
Control
ADU-S100
CTLA-4
Vinculin
CTLA-4/ Vinculin
ns

Supplemental Figure 8. Combination treatment of STING agonist plus αPD-1 and αCTLA-4 antibodies does not further decrease MPNST volume compared to STING agonist plus αPD-1 treatment.

(**A**) Schema of ADU-S100 plus αPD-1 and αCTLA-4 treatment protocol in *cisNP* mice. (**B**) Tumor volume changes in response to the indicated treatments. (**C**) Percentage increase in tumor volume upon indicated treatments. Data sets for ADU-S100 and ADU-S100 plus αPD-1 used in this graph are borrowed from Figure 4D for clarity and ease of comparison. ADU-S100 + αCTLA-4, n=3; ADU-S100 + αPD-1 + αCTLA-4, n=3. (**D**) Western blot analysis for expression of CTLA-4 in MPNSTs harvested from *cisNP* mice treated with vehicle control (n=3) or ADU-S100 (n=3). Quantified protein band intensities are shown graphically on the right. Data are represented as mean ± SEM and p values are determined by Tukey's multiple comparisons test in (C) and two-tailed t test in (D) as indicated. ns = not significant.

A

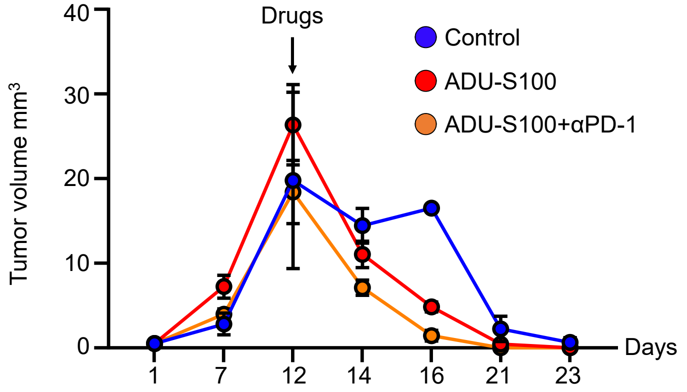

B

	Control vs ADU-S100	Control vs ADU-S100 + αPD-1	ADU-S100 vs ADU-S100 + αPD-1
Day 14	ns	p=0.0169	ns
Day 16	p<0.0001	p<0.0001	p=0.0081

C

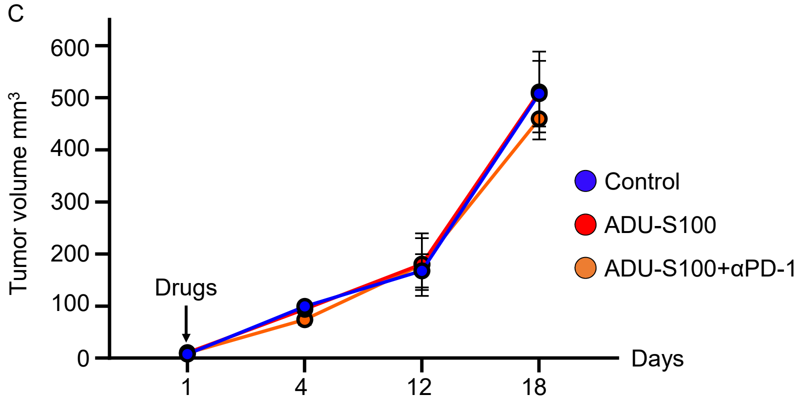

D

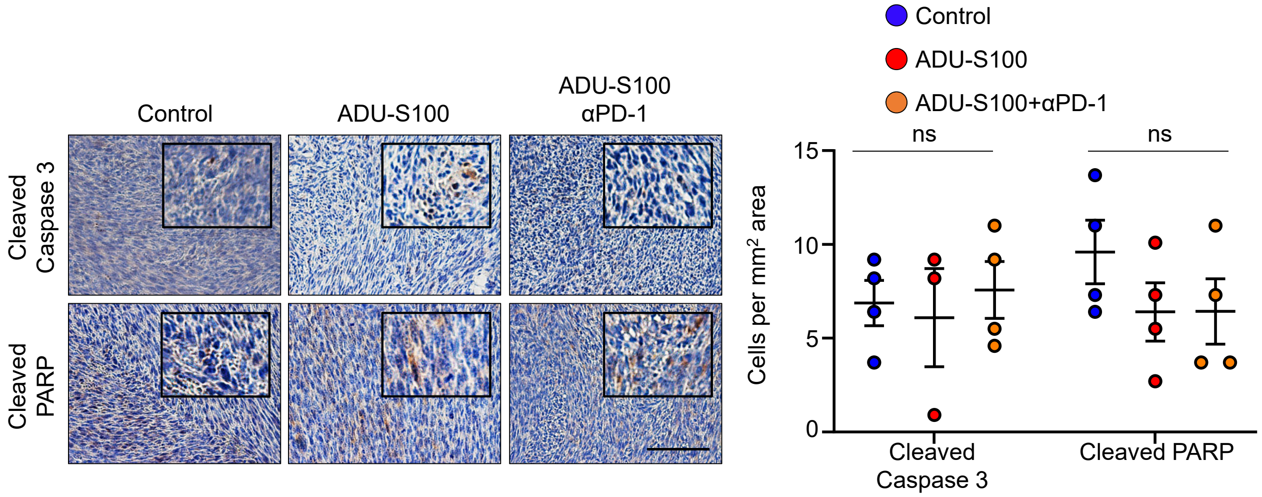

E

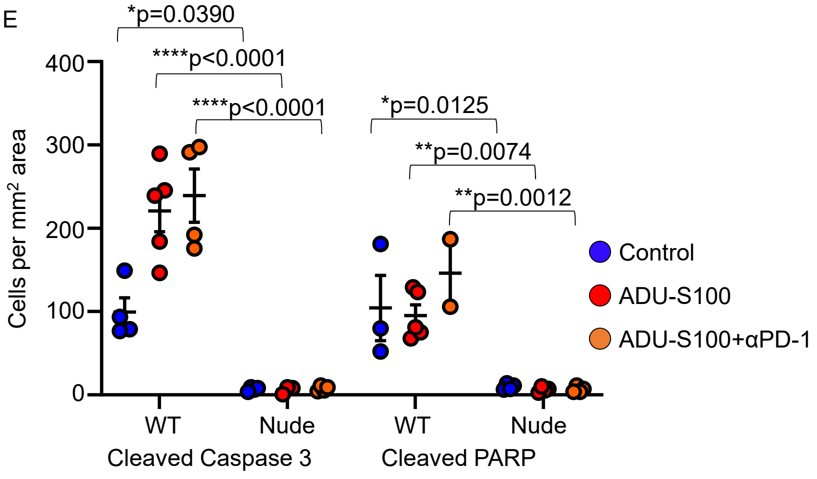

Supplemental Figure 9. Combination treatment of STING activation plus ICB accelerates tumor regression in a human MPNST xenograft mouse model. (**A**) Difference in tumor volume in human MPNST xenograft mice upon treatment with PBS (n=15), ADU-S100 (n=15), or ADU-S100 + αPD-1 (n=15). (**B**) Statistical significance of the volume differences in (A) determined by multiple unpaired t tests. (**C**) Difference in tumor volume in human MPNST xenograft in nude mice upon treatment with PBS (n=4), ADU-S100 (n=4) or ADU-S100 + αPD-1 (n=4). (**D**) Paraffin sections from nude mouse xenograft MPNST treated as indicated were stained for Cleaved Caspase 3 and Cleaved PARP. (**E**) Comparison of Cleaved Caspase 3 and Cleaved PARP levels in wild-type mouse (repeated from data in Figure 6D) and nude mouse xenograft MPNST. Data are represented as mean ± SEM and p values are determined by Tukey's multiple comparisons test as indicated. Scale bar: 50 µm. *$P < 0.05$, **$P < 0.01$, ***$P < 0.001$, ns = not significant.

Supplemental Table 1: Primer sequences for qRT-PCR

Ifnb1	mIfnb1-F1	5’ CCC TAT GGA GAT GAC GGA GA 3’
	mIfnb1-R1	5’ ACC CAG TGC TGG AGA AAT TG 3’
Tnf	mTnf-F1	5’ ACG GCA TGG ATC TCA AAG AC 3’
	mTnf-R1	5’ GTG GGT GAG GAG CAC GTA GT 3’
Cxcl10	mCxcl10-F1	5’ GCT GCA ACT GCA TCC ATA TC 3’
	mCxcl10-R1	5’ GTG GCA ATG ATC TCA ACA CG 3’
Il12a	mIl12a-F1	5’ CTC CTG TGG GAG AAG CAG AC 3’
	mIl12a-R1	5’ CAG ATA GCC CAT CAC CCT GT 3’
