# Supplementary material for: STING activation reprograms the microenvironment to sensitize NF1-related malignant peripheral nerve sheath tumors for immunotherapy
Source: J Clin Invest. 2024 Mar 19;134(10):e176748. doi: 10.1172/JCI176748 (PMC11093615; doi:10.1172/JCI176748)

Unedited Blots and Gels

Figure 2, panel B

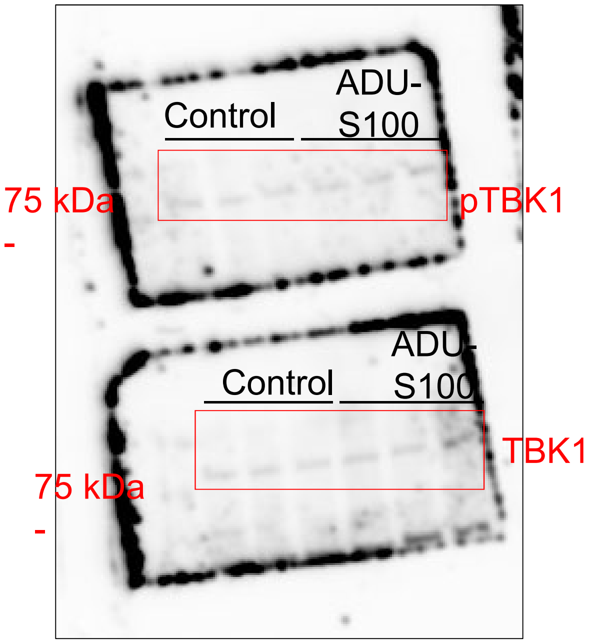

Figure 2, panel B

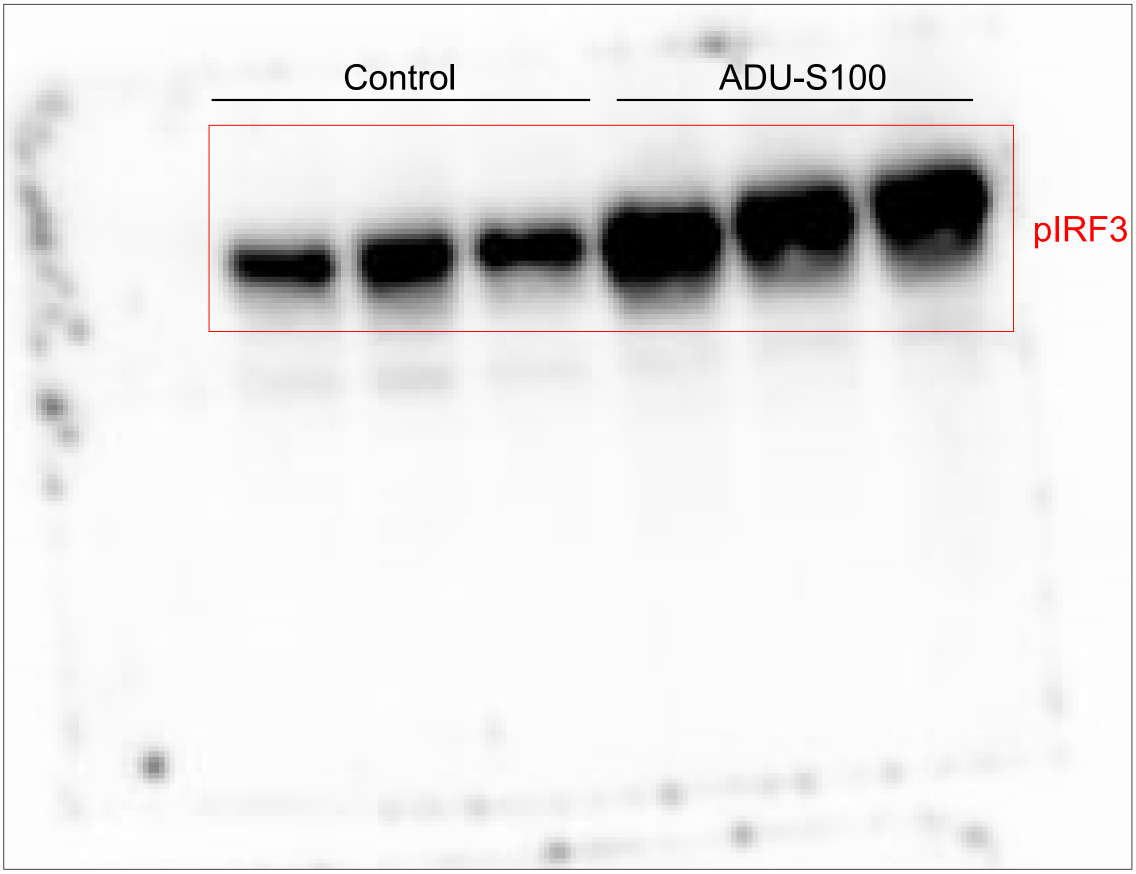

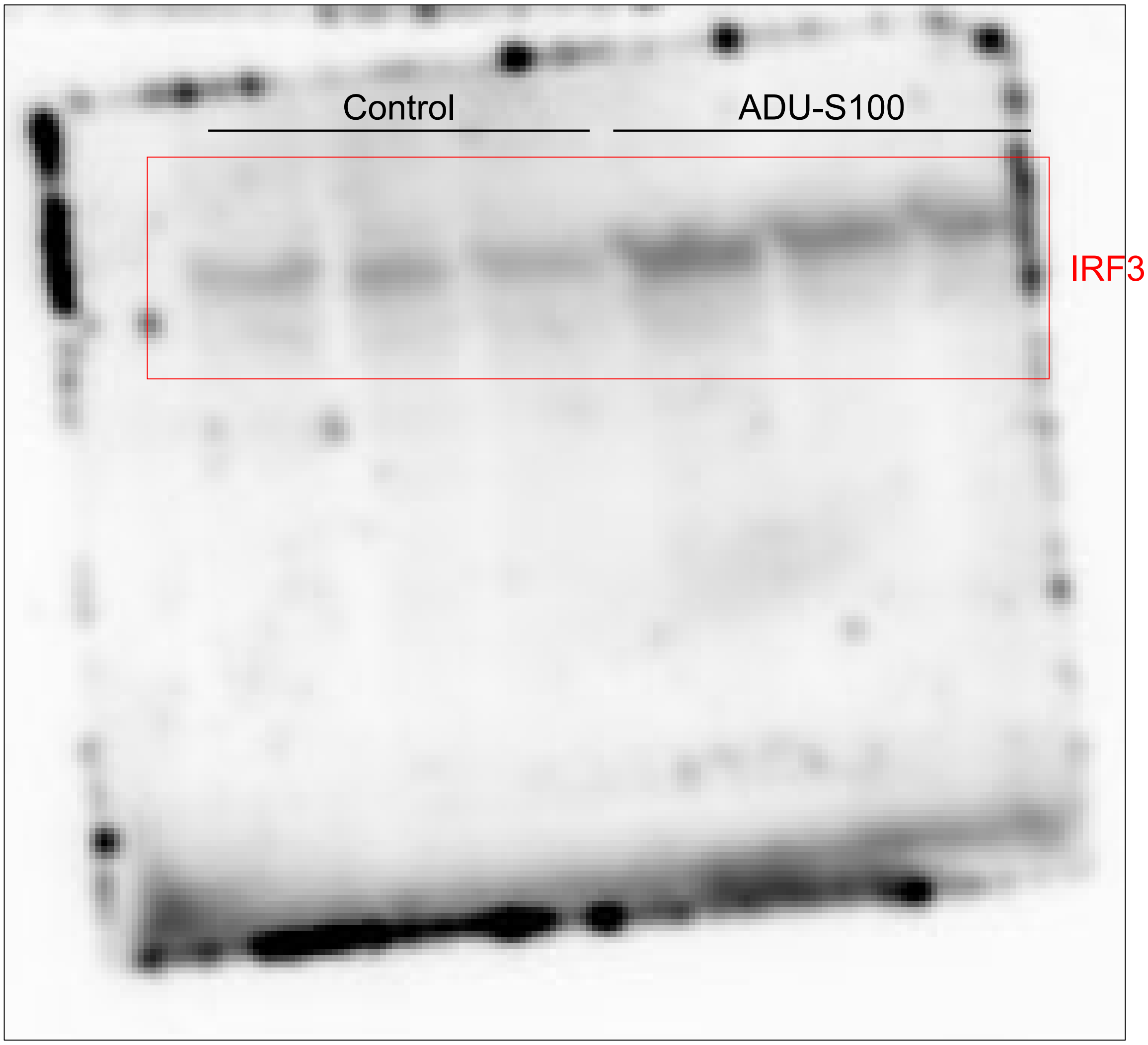

Figure 2, panel B

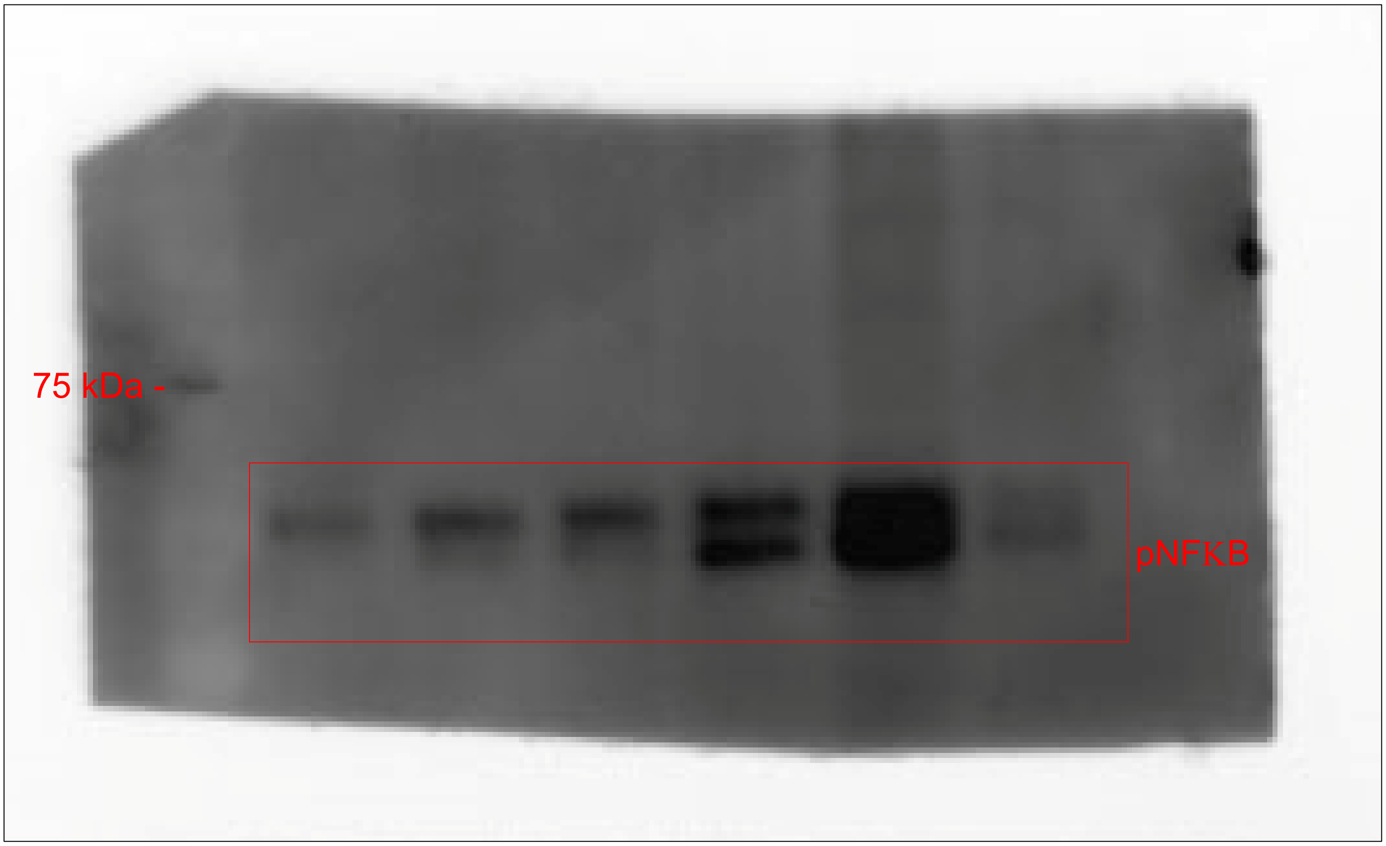

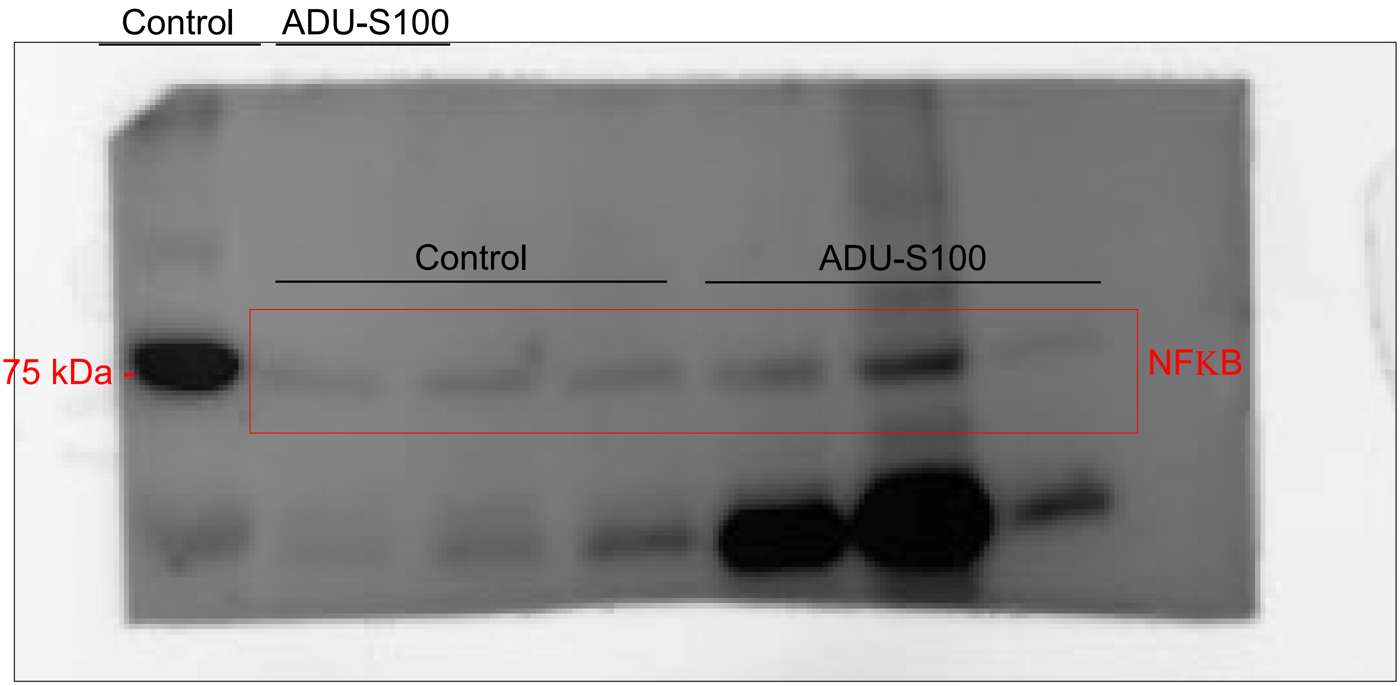

Figure 2, panel B

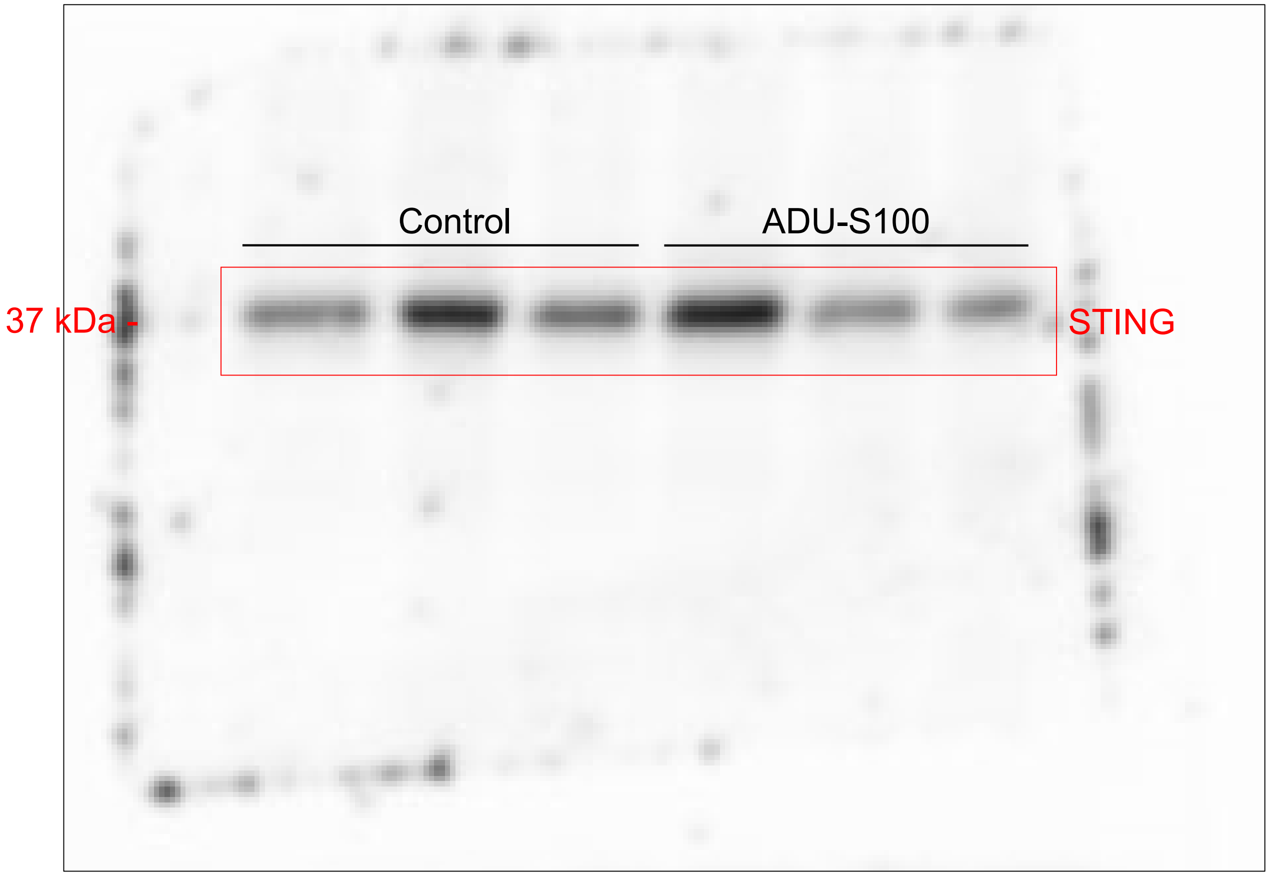

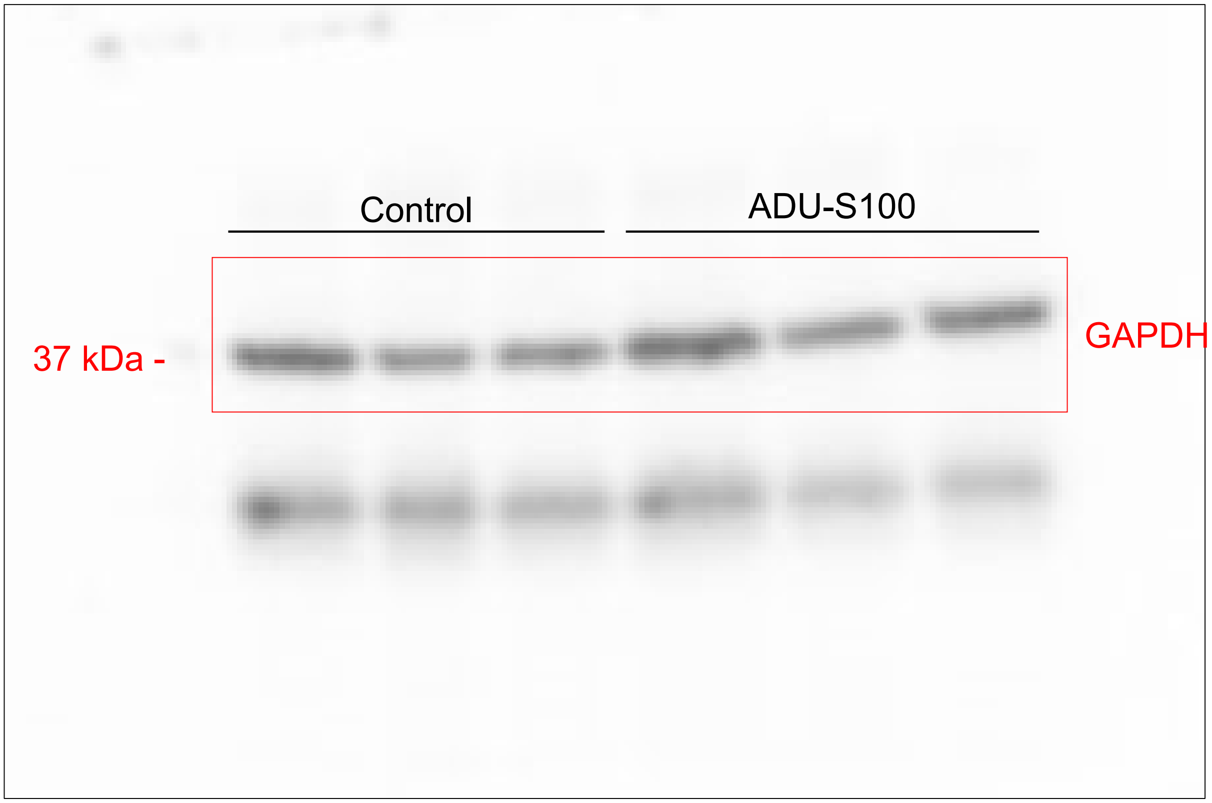

Figure 2, panel D

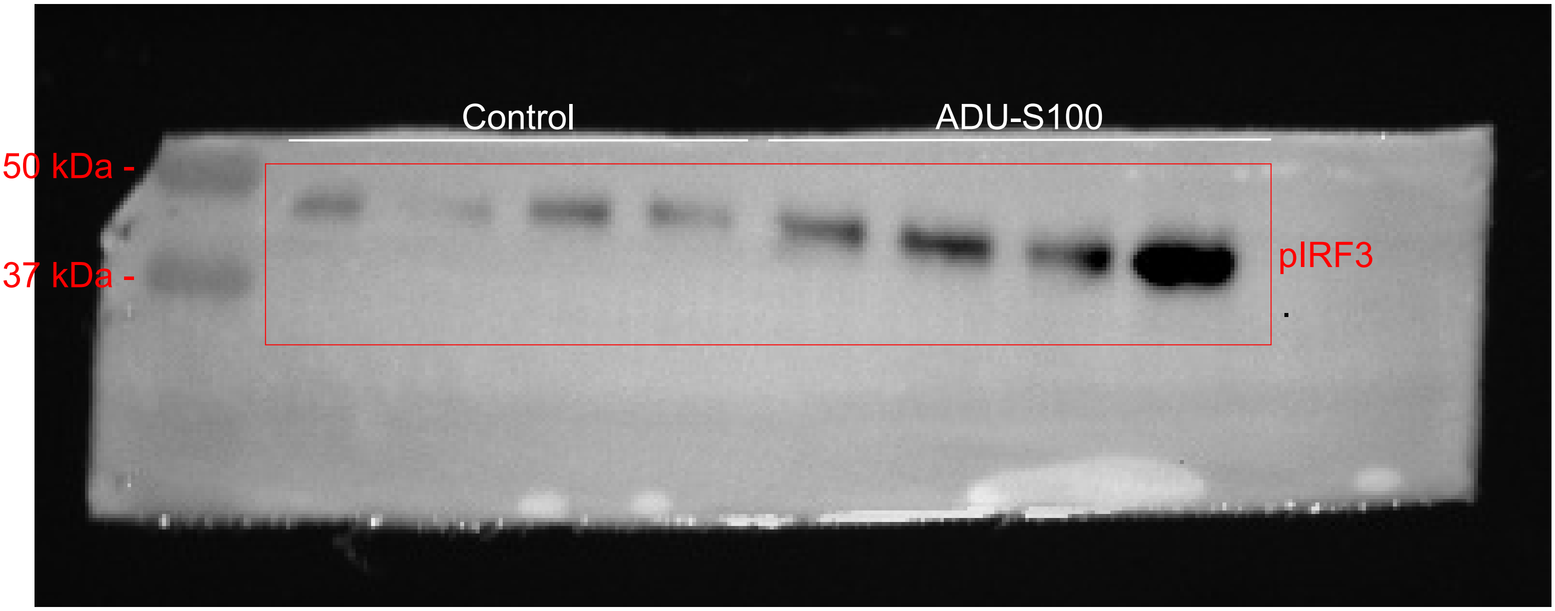

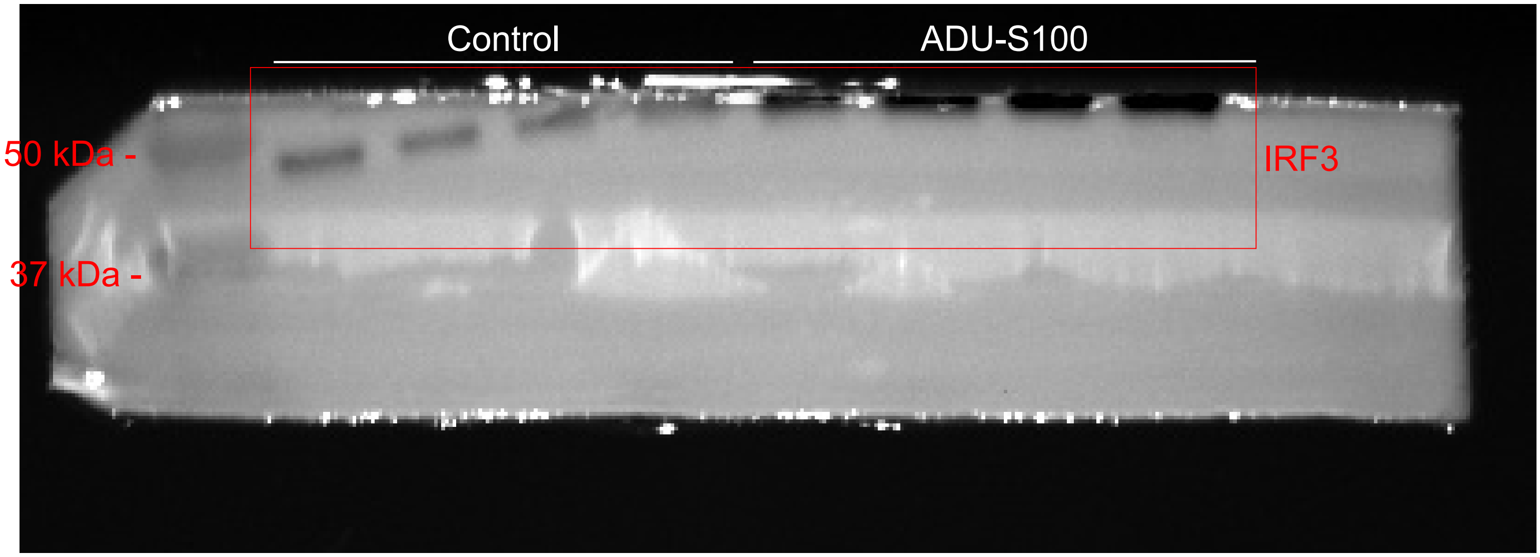

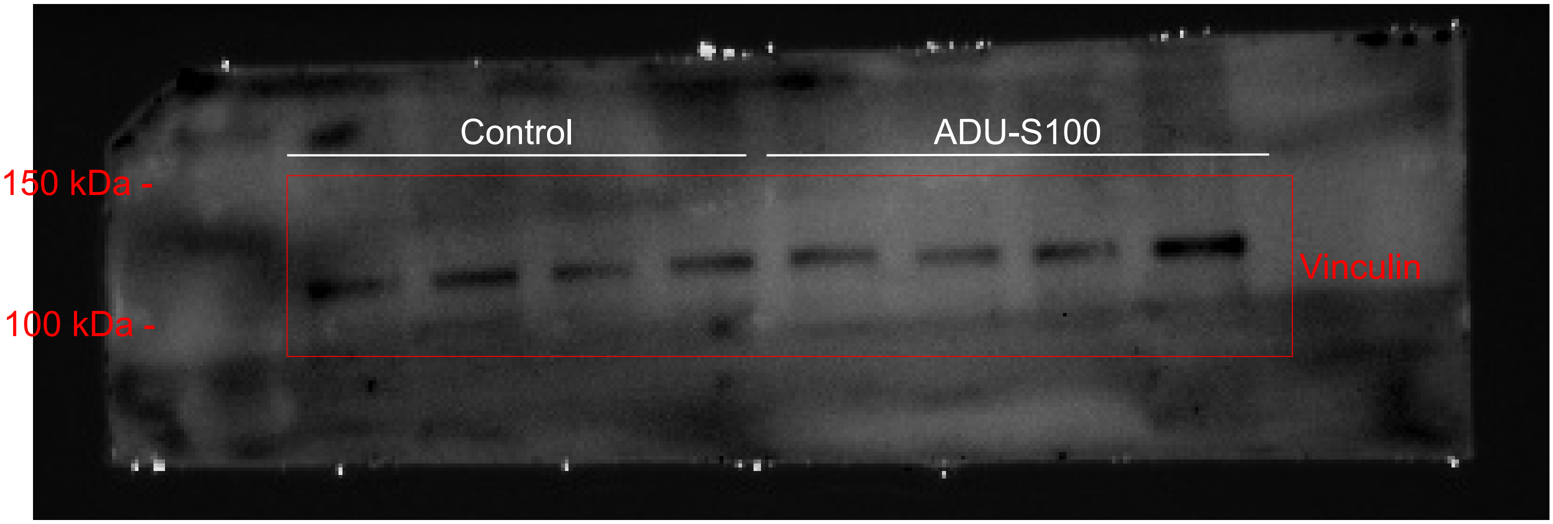

Supplemental Figure 1, panel A

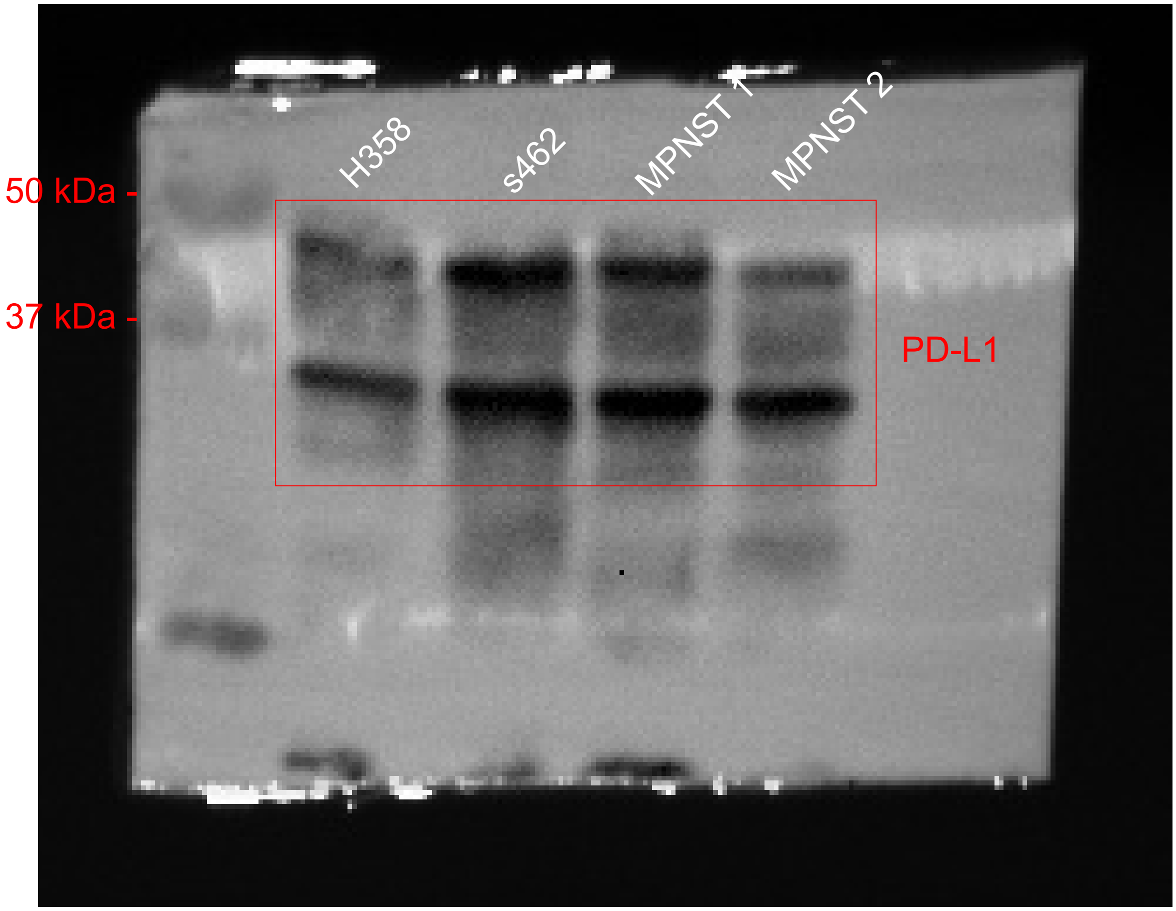

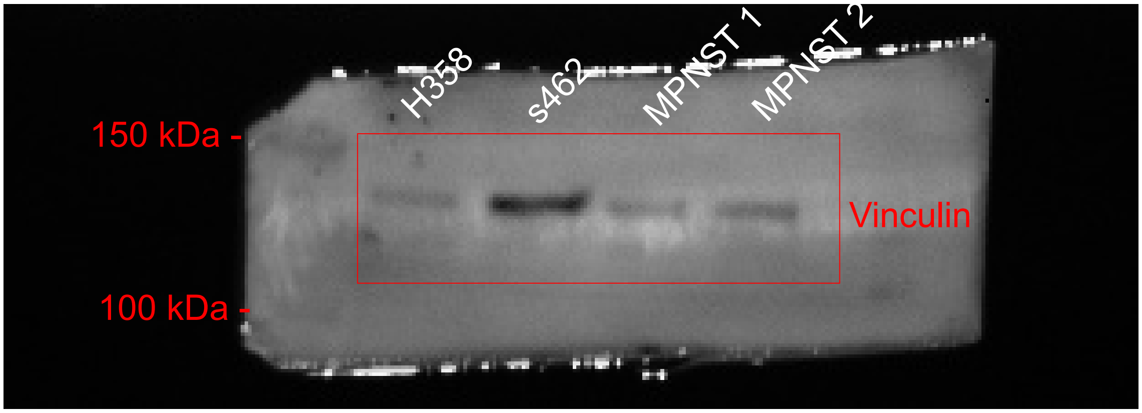

Supplemental Figure 1, panel B

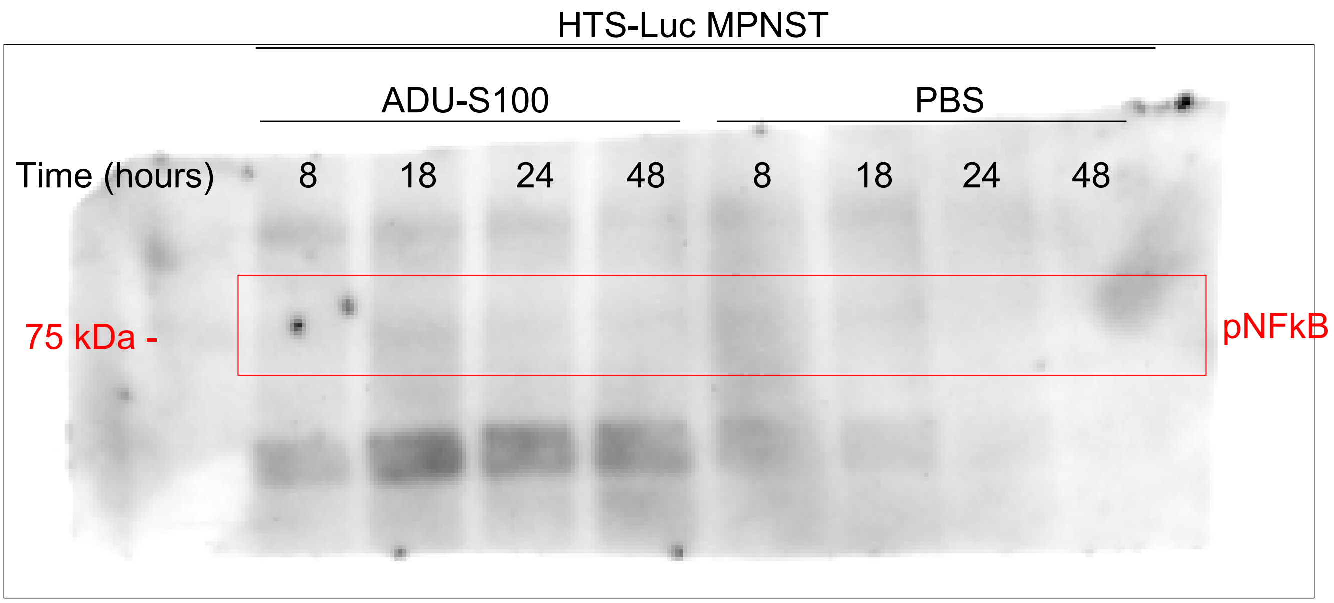

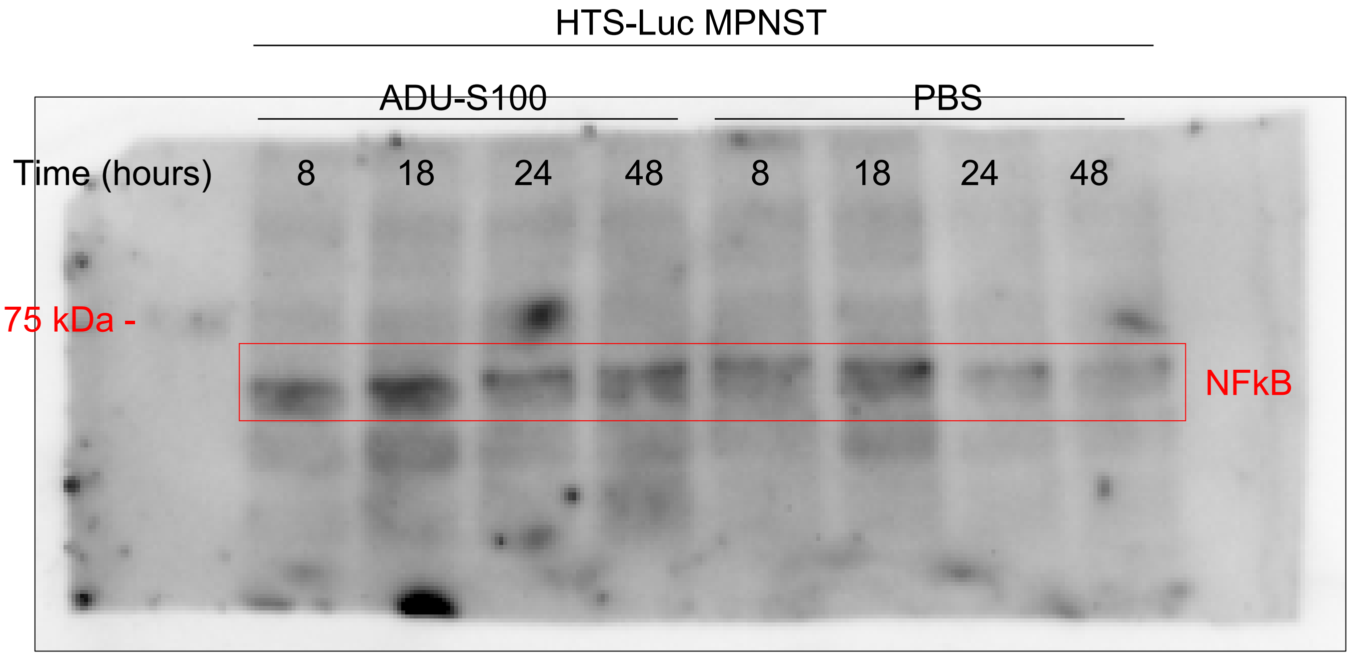

Supplemental Figure 1, panel B

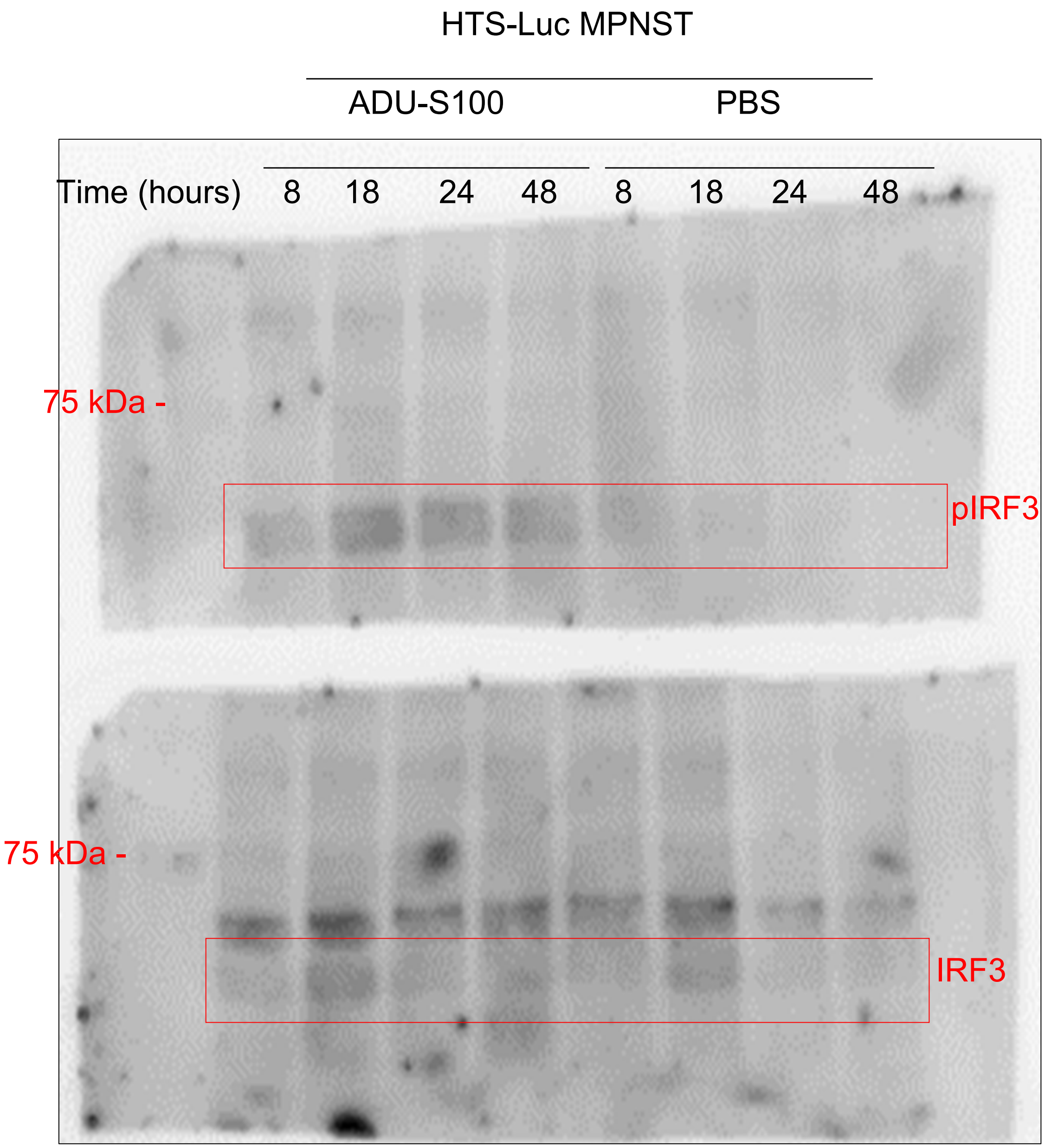

Supplemental Figure 1, panel B

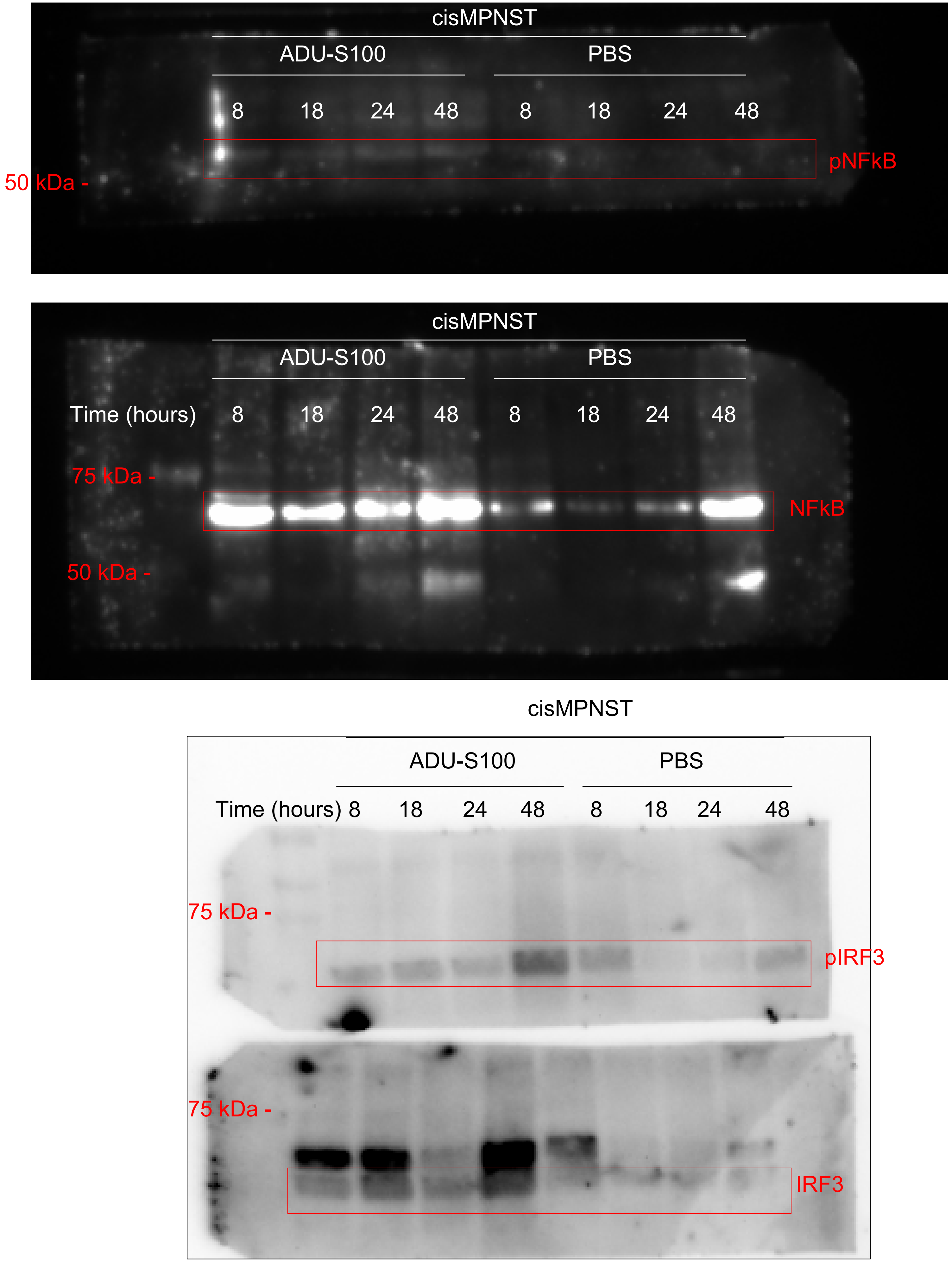

Supplemental Figure 2, panel B

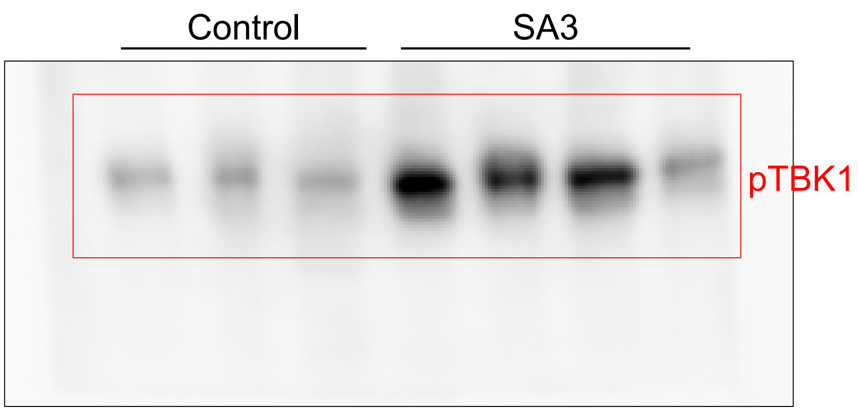

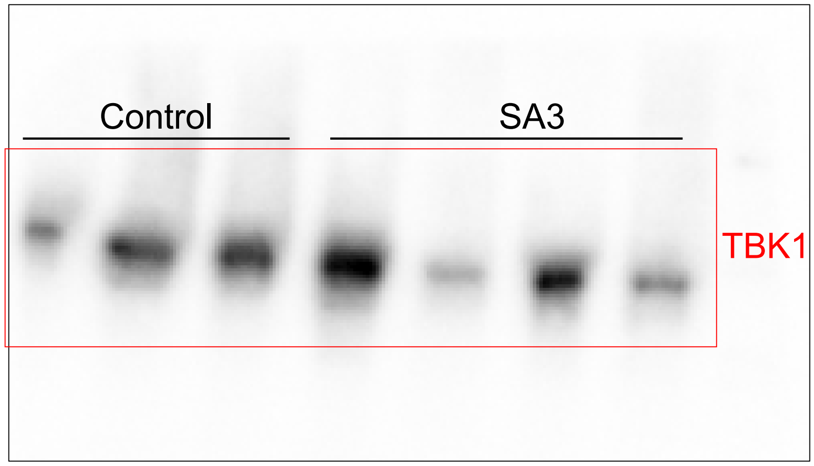

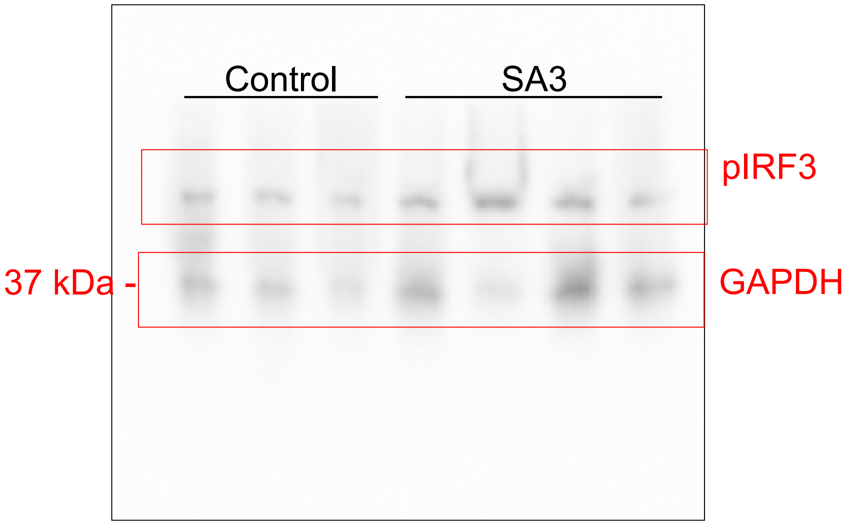

Supplemental Figure 2, panel B

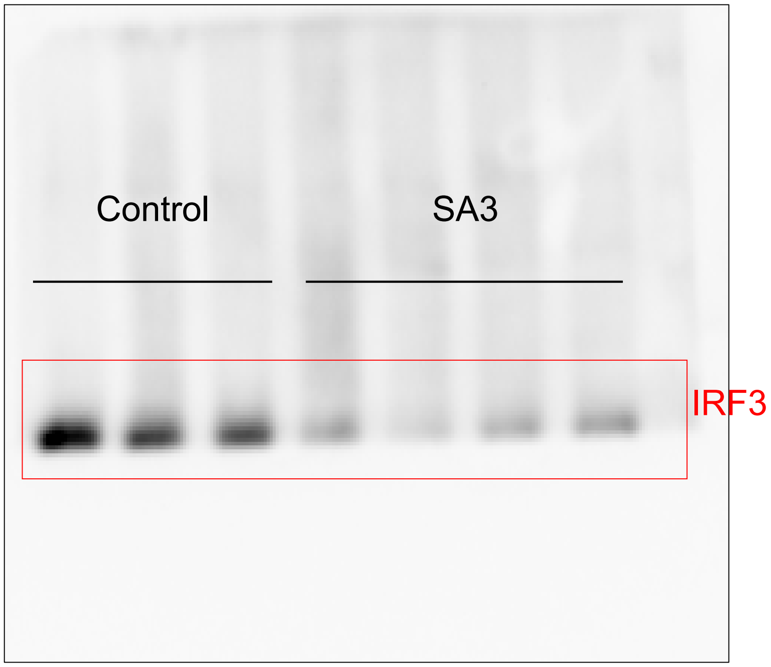

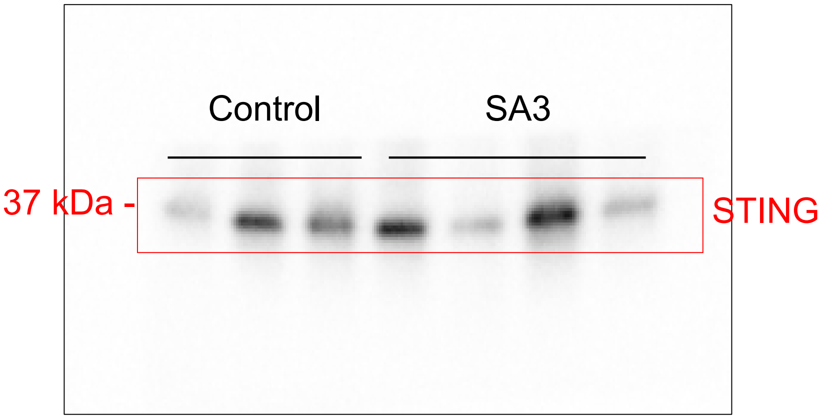

Supplemental Figure 3, panel B

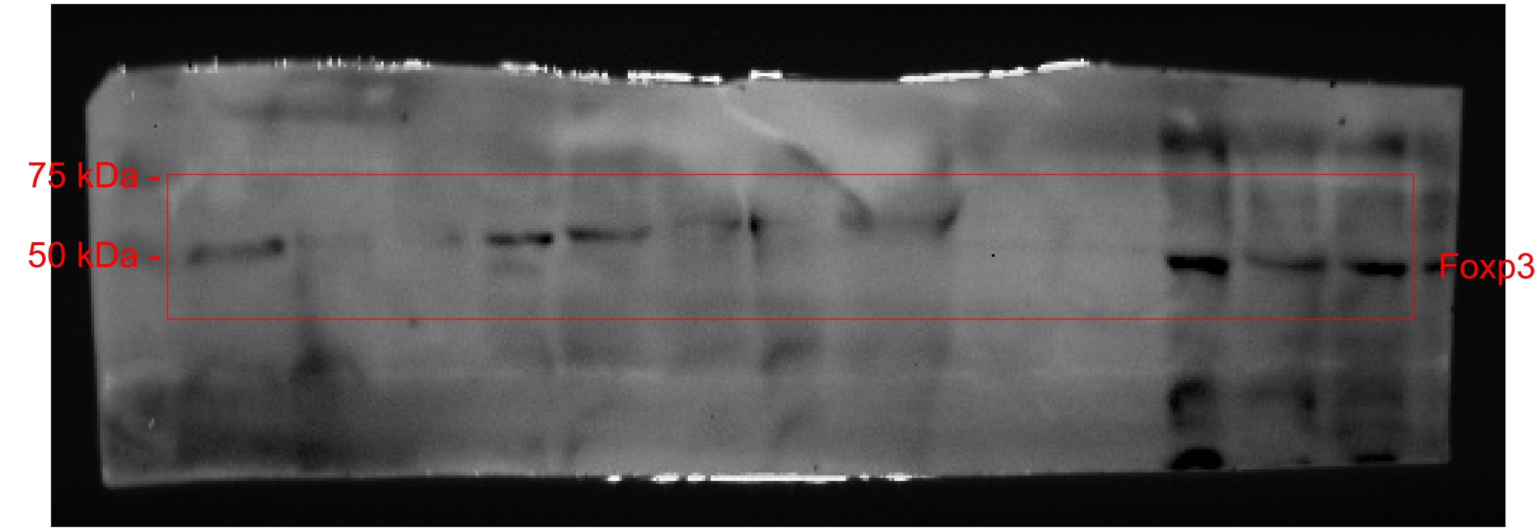

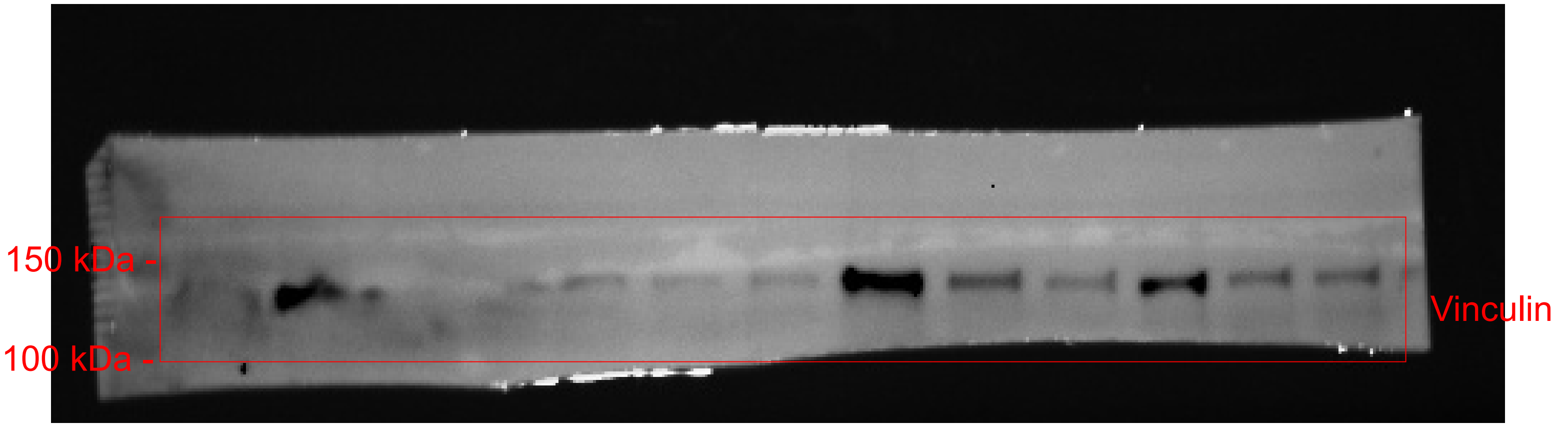

Supplemental Figure 4, panel D

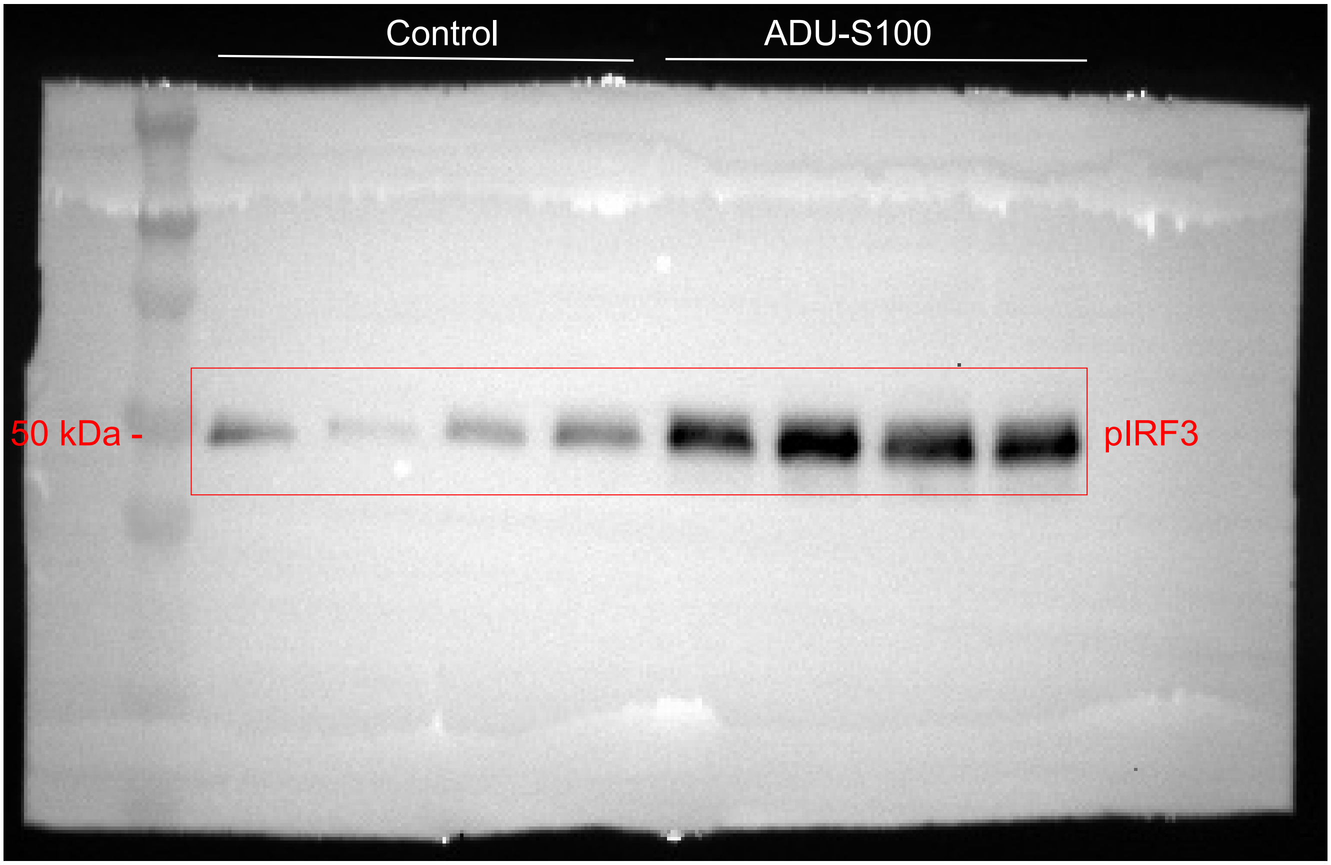

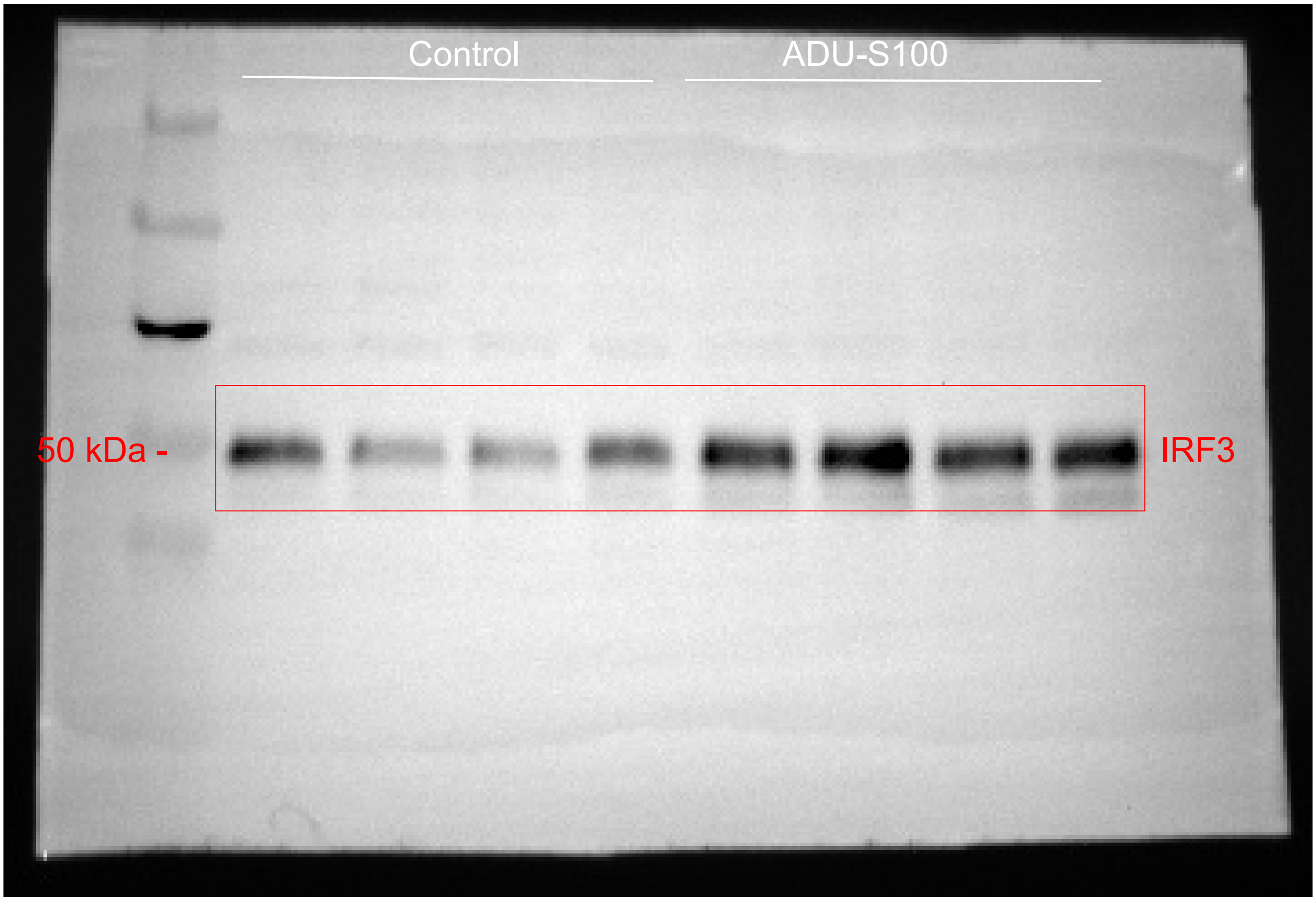

Supplemental Figure 4, panel D

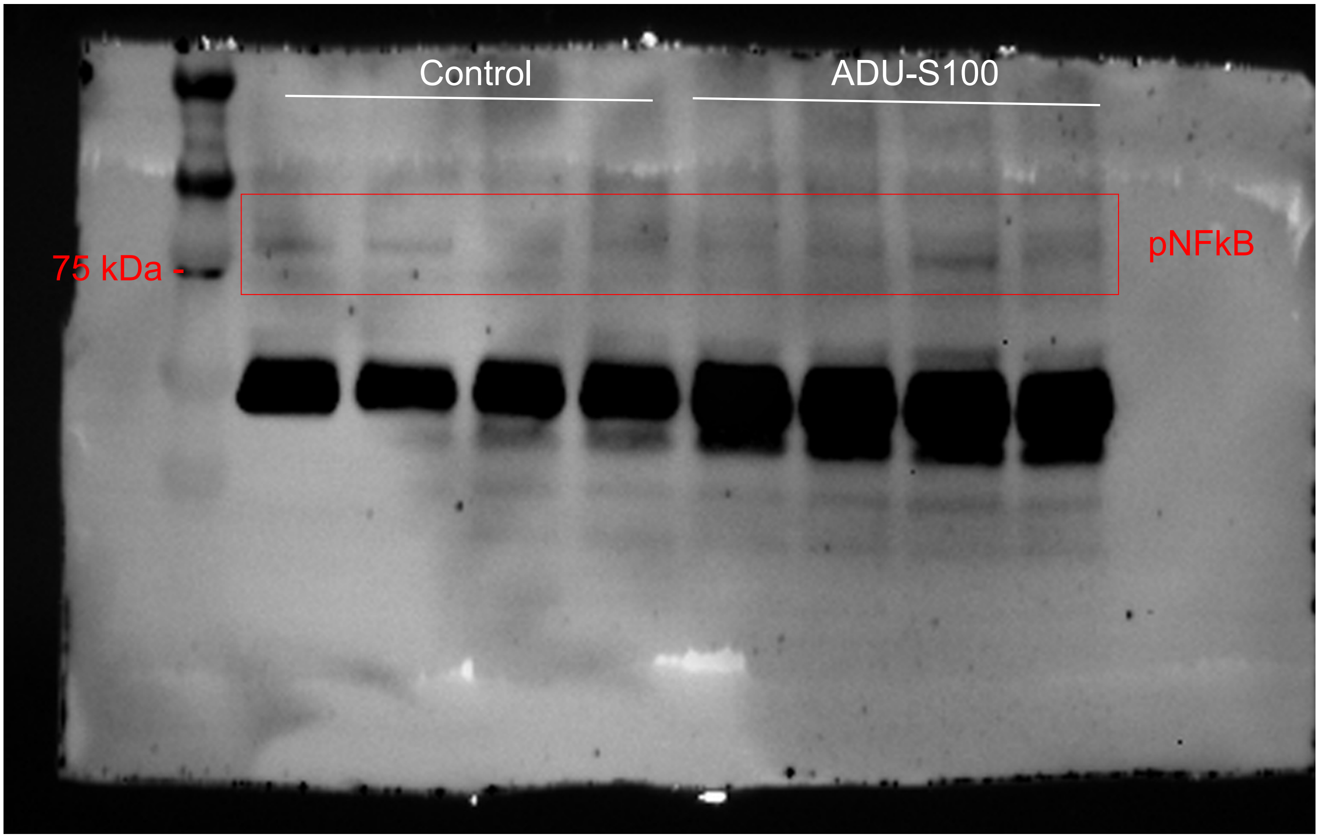

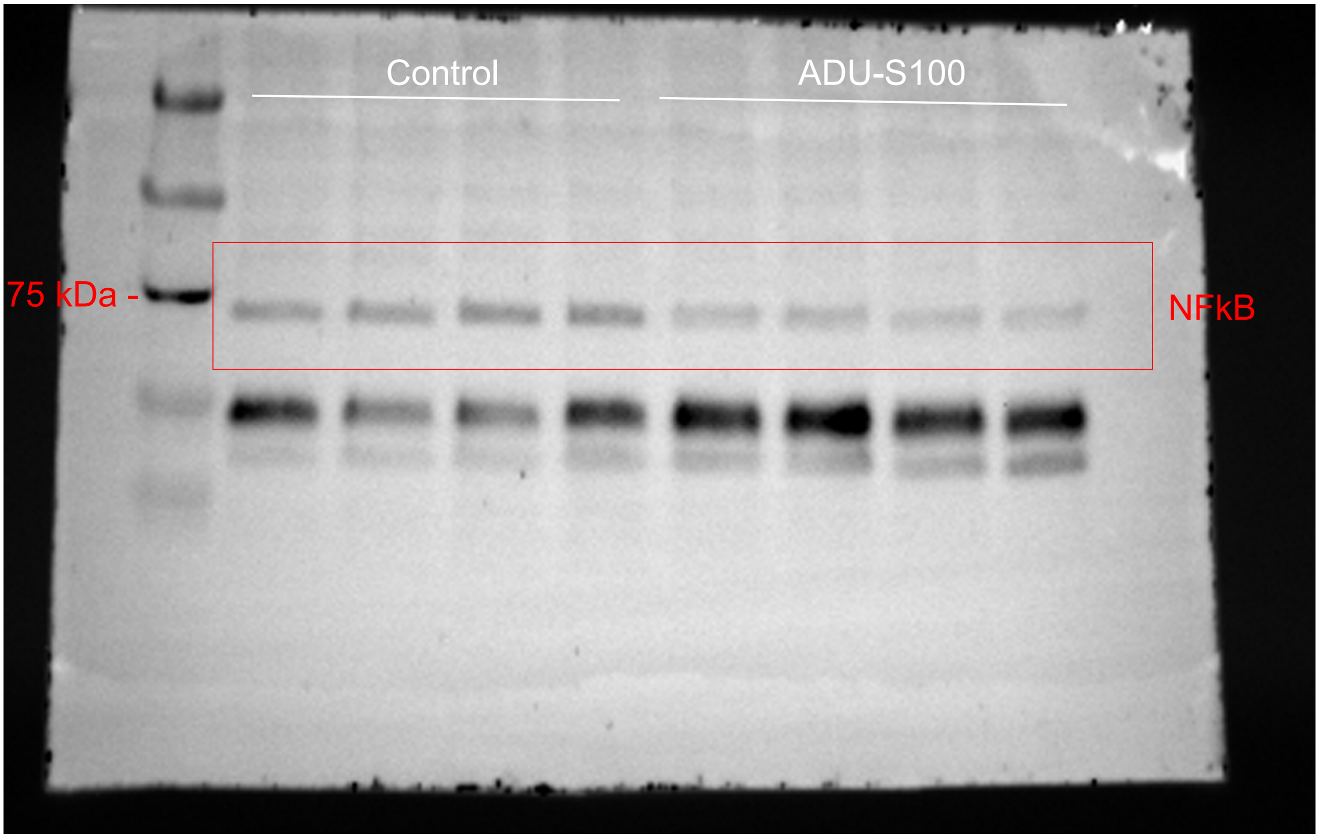

Supplemental Figure 4, panel D

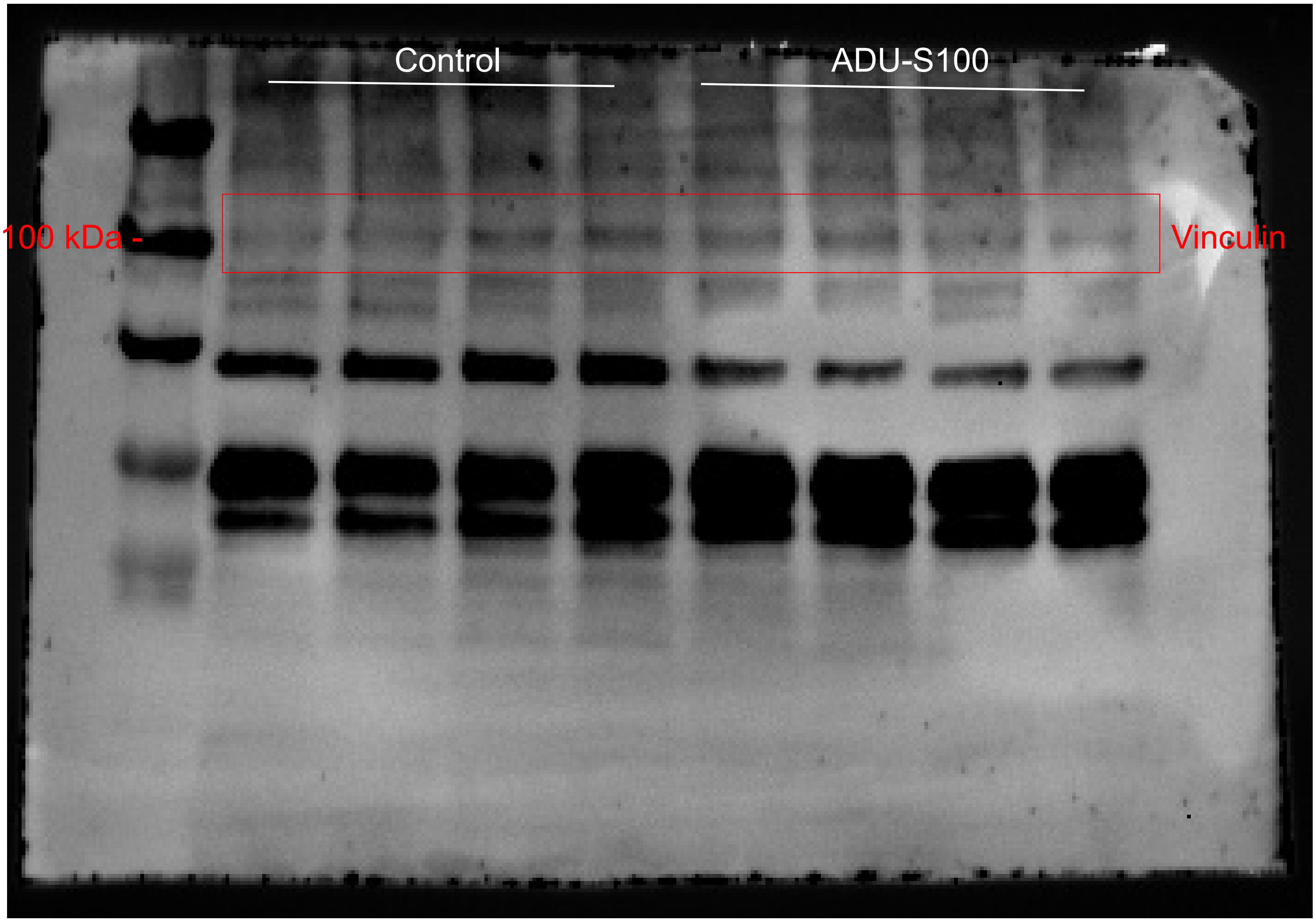

Supplemental Figure 8, panel D

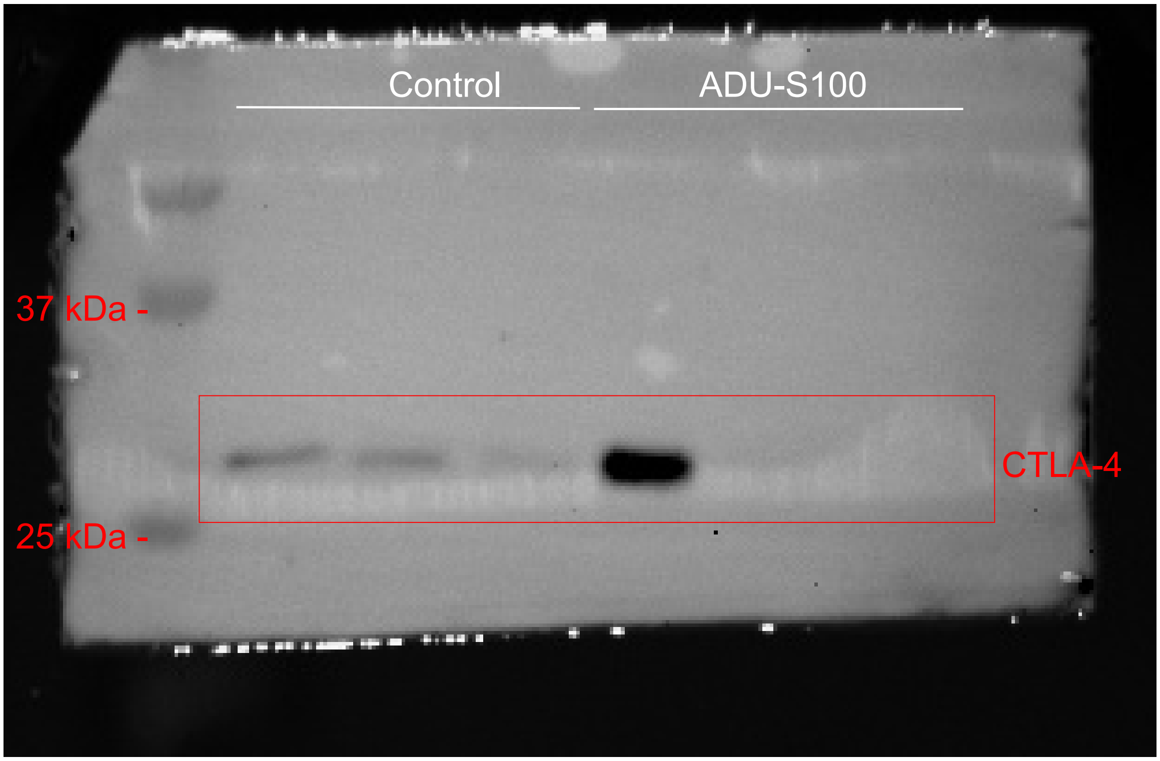

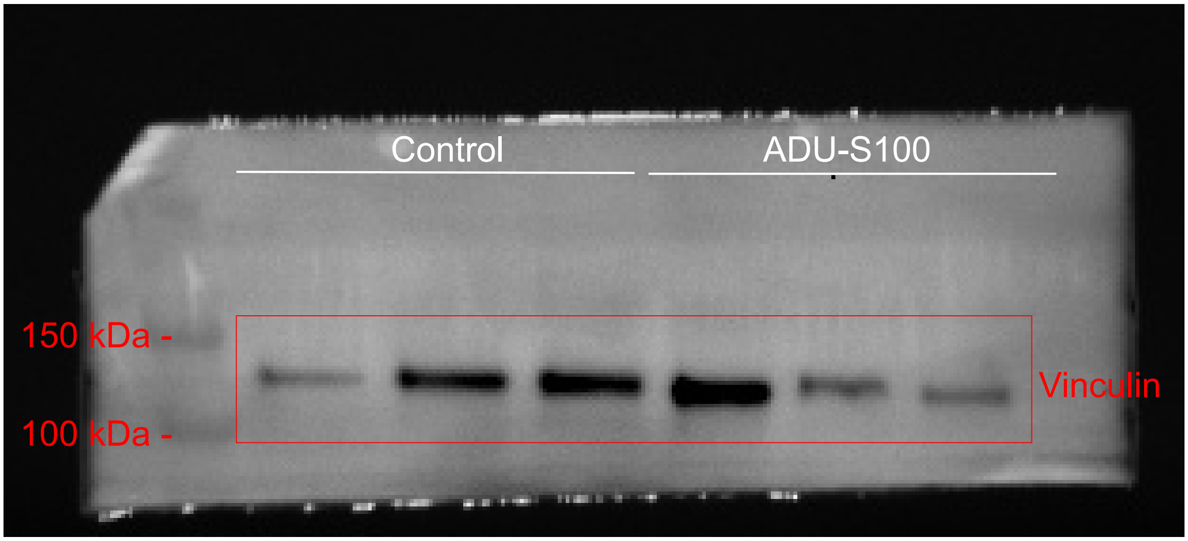

Supplement: Unedited blot and gel images [file jci-134-176748-s216.pdf]
